# Resolved genomes of wastewater ESBL-producing *Escherichia coli* and metagenomic analysis of source wastewater samples

**DOI:** 10.1128/spectrum.00717-24

**Published:** 2024-08-21

**Authors:** Clinton Cheney, Jared D. Johnson, John P. Ste. Marie, Kayla Y. M. Gacosta, Natalie B. Denlinger Drumm, Gerrad D. Jones, Joy Waite-Cusic, Tala Navab-Daneshmand

**Affiliations:** 1 School of Chemical, Biological, and Environmental Engineering, Oregon State University, Corvallis, Oregon, USA; 2 Department of Food Science and Technology, Oregon State University, Corvallis, Oregon, USA; 3 Department of Biological and Ecological Engineering, Oregon State University, Corvallis, Oregon, USA; Dominican University New York, Orangeburg, New York, USA

**Keywords:** antimicrobial resistance, antibiotic resistance, wastewater treatment, *Escherichia coli*, extended-spectrum beta-lactamases

## Abstract

Extended-spectrum beta-lactamase (ESBL)-producing *Escherichia coli* pose a serious threat to human health because of their resistance to the most commonly prescribed antibiotics: penicillins and cephalosporins. In this study, we provide a genomic and metagenomic context for the determinant beta-lactam resistance genes of ESBL-positive *E. coli* isolated from various wastewater treatment utilities in Oregon, USA. Class A beta-lactamase genes on chromosomes (*bla*CTX-M, *bla*TEM) were clustered with antibiotic resistance genes associated with other classes of antibiotics (sulfonamides and aminoglycosides) along with insertional elements. ESBL genes such as *bla*CTX-M, *bla*TEM, and *bla*SHV were also detected on conjugable plasmids of IncF and IncI incompatibility types. One novel IncF plasmid (pSHV2A_ESBLF) was identified, which carried a multidrug resistance genotype (*bla*SHV-2A, *aadA22*, *aac*3, *aph*6, *tet*A, and *sul*1) in addition to a *mer* (mercury resistance) operon, colicin, and aerobactin genes. Shotgun metagenomic analysis of the ESBL-producing *E. coli*-originating wastewater samples showed the presence of class A beta-lactamases; however, the ESBL genes identified in the *E. coli* genomes were below the detection limits. Other ESBL-associated genes (i.e., *bla*OXA.11, *bla*FOX.7, and *bla*GES.17) were identified in the wastewater samples, and their occurrences were correlated with the core microbial genera (e.g., *Paraprevotella*). In the *E. coli* genomes and wastewater samples, tetracycline, aminoglycoside, and beta-lactam resistance determinants frequently co-occurred. The combination of whole-genome and metagenomic analysis provides a holistic description of ESBL-producing organisms and genes in wastewater systems.

**IMPORTANCE:**

Using a hybrid sequencing and assembly strategy (short- and long-read sequencing), we identified the distribution of ARGs and virulence factors harbored on plasmids and chromosomes. We further characterized plasmids’ incompatibility types and the co-occurrences of ARGs and virulence factors on plasmids and chromosomes. We investigated the transferability of plasmid-mediated beta-lactams via conjugation. Finally, using shotgun metagenomic analysis of the ESBL-producing *Escherichia coli*-originated wastewater samples, we described the microbial community, the resistome composition, and the potential associations with plasmid-mediated beta-lactam genes and other ARGs.

## INTRODUCTION

Antimicrobial-resistant (AMR) pathogens—referred to as the Silent Pandemic—are a major public health concern (1). In 2019, 4.95 million deaths were attributed to AMR pathogens globally, with the virulent *Escherichia coli* as the leading cause of these deaths (2). The U.S. Centers for Disease Control and Prevention has distinguished extended-spectrum beta-lactamase (ESBL)-producing Enterobacterales as one of the most serious threats facing humanity’s efforts against AMR (3). ESBL-associated genes confer resistance to a broad spectrum of the most commonly prescribed antibiotic class: beta-lactams, including penicillins and first- to third-generation cephalosporins (4, 5).

ESBL-producing *E. coli* may be associated with multidrug resistance (MDR), a classification of resistance to three or more classes of antibiotics (6). Moreover, antibiotic resistance genes (ARGs), including beta-lactam genes, can be carried on plasmids, which play a major role in the dissemination of AMR in the environment via the horizontal gene transfer (HGT) mechanism of conjugation (6–8). IncF plasmids, which have a narrow host range of Enterobacteriaceae and are common within *E. coli* genomes, have been documented to carry *bla*CTX-M-type ESBL genes (9, 10). Beta-lactam-associated IncF plasmids have also been described as MDR, either carrying determinants for cross-resistant efflux pumps (i.e., *mac*B, *Emr*B, and *Mdt*K) or harboring multiple classes of ARGs, providing the hosting *E. coli* strain with resistance to multiple antibiotics, such as aminoglycosides, macrolides, and tetracyclines (11, 12). The prevalence of beta-lactam-associated genes on plasmids alongside resistance to other antibiotic classes as well as antimicrobials such as metals informs the risks associated with the potential transfer of these plasmids from the host bacteria to other microbial communities.

Beta-lactam-encoding genes are common in nature, having been found in bacterial isolates and environmental samples on all seven continents, including remote areas in Antarctica (13–19). Municipal wastewater and wastewater treatment utilities are important reservoirs for the prevalence and dissemination of ARGs. The beta-lactam genes within the class-A beta-lactamase family (*bla*CTX-M, *bla*TEM, and *bla*SHV) are commonly detected in wastewater according to a recent review (20). A 2019 survey of ESBL-producing *E. coli* isolated from wastewater in the United States showed a concerning rate of resistant phenotypes to third-generation cephalosporins, carrying *bla*TEM beta-lactam genes, as well as the carbapenemase *bla*VIM (21). Co-localization of virulence factors and beta-lactams on the same plasmid have been reported in isolates from wastewater samples (22, 23). This co-localization is a concern because of the association between pathogenicity and limited clinical treatment options. With a highly diverse set of bacteria in biological processes such as activated sludge, the transfer of ARGs between a variety of bacteria could support the proliferation of AMR in effluent water and biosolids streams (24, 25). Given the serious threat of ESBL-producing *E. coli* and the link between wastewater and public health, further characterization of wastewater bacteria and the resistome associated with wastewater streams is necessary to inform risk assessment and subsequent policymaking.

In this article, we resolved the genome of ESBL-producing *E. coli* isolates collected from several wastewater utilities whose AMR genotypes and phenotypes were previously characterized in our lab (26). Using a hybrid sequencing and assembly strategy (short- and long-read sequencing), we identified the distribution of ARGs and virulence factors harbored on plasmids and chromosomes. We further characterized plasmids’ incompatibility types and the co-occurrences of ARGs and virulence factors on plasmids and chromosomes. We investigated the transferability of plasmid-mediated beta-lactams via conjugation. Finally, using shotgun metagenomic analysis of the ESBL-producing *E. coli*-originated wastewater samples, we described the microbial community, the resistome composition, and the potential associations with plasmid-mediated beta-lactam genes and other ARGs.

## MATERIALS AND METHODS

### *E. coli* isolates and wastewater samples

*E. coli* strains were previously isolated from influent, secondary effluent, final effluent, and biosolids streams of eight wastewater treatment utilities across Oregon as described in our previous study (Table S1) (26). Wastewater samples were collected between January 2019 and September 2020. For liquid samples (i.e., influent, secondary effluent, and final effluent), incremental volumes were filter concentrated through a 0.45-µm mixed-cellulose ester membrane (Whatman, Kent, UK) until the filter clogged. The filtered volumes were up to 40 mL for influent and up to 400 mL for secondary and final effluents. The filter paper was fixed with 1 mL of 50% ethanol and stored at −20°C until further processing. For biosolids, between 0.25 and 0.50 g of biosolids were transferred to sterile microcentrifuge tubes and stored at −20°C until further processing.

### Whole genome sequencing and analysis

Frozen stock cultures of *E. coli* isolated from the wastewater samples were streaked onto tryptic soy agar (Hardy Diagnostics, Santa Maria, CA) and grown for 24 hours at 37°C before being transferred to tryptic soy broth (TSB, Hardy Diagnostics, Santa Maria, CA) and cultured under the same conditions. DNA was extracted from TSB cultures following the manufacturer’s instructions using the DNeasy Blood and Tissue Kit (Qiagen, Carlsbad, CA). Purified DNA was quantified and quality checked using the Qubit 4 (Invitrogen, Carslbad, CA) and NanoDrop One Micro UV-VIS Spectrophotometer (Thermo Fisher Scientific, Waltham, MA). Long-read sequencing libraries were prepared using the Rapid Barcoding Sequencing Kit (SQK-RBK004) and sequenced on a MinION system (Oxford Nanopore, Oxford, UK) following the manufacturer’s protocol. Short-read sequencing was performed using the Illumina MiSeq as described previously (26).

Hybrid assembly of MinION and Illumina reads was performed with Trycycler v0.5.0 and Flye v2.9 for each isolate (27, 28). Chromosomes were defined as circularized contigs with lengths of 4.6–5.0 Mbp, and plasmids were defined as circularized extrachromosomal contigs. BLAST alignment analysis was performed and visualized with BRIG (29). Chromosome and plasmid contigs were annotated with Prokka v1.14.5 for general annotation of coding sequences, and NCBI’s AMRFinderPlus v3.10 and the CGE’s VirulenceFinder v2.0 were used to identify ARGs and virulence genes, respectively (30–32). Plasmid contigs were submitted to the pMLST web tool to determine associations with plasmid incompatibility groups (33).

### Conjugation assays

The nine resolved ESBL-positive *E. coli* isolates (with the circularization of chromosomes and plasmids) in the study were used as donor strains in broth-mating conjugation assays with sodium azide-resistant *E. coli* J53 (ATCC BAA-2731, ATCC, Manassas, VA) as the recipient strain. Overnight cultures were grown at 37°C in 50 mL Luria Broth (LB) supplemented with 5 mg/mL of cefotaxime (CTX) for donors and 100 mg/mL of sodium azide for the recipient. Overnight cultures were centrifuged at 4,500 × *g* for 15 minutes and resuspended in phosphate-buffered saline (PBS) twice to remove excess antibiotics. Donor and recipient cultures were mixed in equal proportions to a final volume of 1 mL before co-incubation for 1 hour at 22°C. After incubation, mixtures were serially diluted in PBS and 70 µL of dilutions was spread on MacConkey agar with MUG plates (Hardy Diagnostics, Santa Clara, CA) containing 5 mg/mL CTX and 100 mg/mL sodium azide. The plates were incubated overnight at 37°C to isolate transconjugant colonies. Six presumptive transconjugant colonies were randomly selected and grown overnight in 1 mL of LB prior to DNA extraction using the Wizard Genomic DNA Purification Kit (Promega, Madison, WI). The AMR genotypes of transconjugant colonies were determined by polymerase chain reaction assays on a T100 Thermal Cycler (Bio-Rad, Hercules, CA). Reaction volumes were 25 µL and consisted of 12.5 µL of Accustart II PCR Toughmix (Quantabio, Beverly, MA) and 0.2 µM concentration of forward and reverse primers. PCR assays involved an initial denaturation at 3 minutes at 94°C, followed by amplification cycles consisting of 15 s of denaturation at 94°C, 30 s of annealing, and 30 s extension at 72°C for 35 cycles, followed by a final extension of 7 minutes at 72°C. Details of primers used in the study and associated annealing temperature are shown in Table S2. PCR products were viewed using a Gel Doc EZ Imager with Image Lab 5.2.1 software (Bio-Rad, Hercules, CA) on 3% agarose gel (VWR, Radnor, PA) with RedSafe Nucleic Acid Staining Solution (Bulldog Bio, Portsmouth, NH) and electrophoresis settings of 85 V for 45 minutes.

### Metagenomic sequencing and bioinformatics

DNA was extracted from wastewater samples using the FastDNA SPIN Kit for Soil (MP Biomedicals, Irvine, CA) following the manufacturer’s protocols and procedures described previously (34). For liquid samples (i.e., influent, secondary effluent, and final effluent), the stored filter paper was removed from the ethanol solution, torn into small pieces, and transferred to the lysing tube. The remaining ethanol was centrifuged at 5,000 × *g* for 10 minutes, and the supernatant was discarded. The pelleted cells were resuspended in the kit-supplied sodium phosphate buffer and transferred to the lysing tube. The remainder of the extraction process followed the manufacturer’s protocol. For biosolids, the manufacturer’s protocols were followed. DNA concentration and purity were determined using the NanoDrop One. The identifiers (IDs) assigned to the wastewater samples (source IDs) in the metagenomics analysis section correspond to the same letters as those of the *E. coli* isolates associated with those samples (Table S1).

DNA samples were sequenced at the Center for Quantitative Life Sciences at Oregon State University (Corvallis, OR) on an Illumina HiSeq platform using a Nextera XT library preparation kit (Illumina, San Diego, CA). Primer and adaptor removal and read-trimming were performed with fastP using default settings for Nextera adapters (35). Trimmed reads were assembled with MEGAHIT v1.2.9, and taxonomic annotation was performed with Kaiju v1.8.2 (36, 37). Annotation of ARGs in the samples was performed using the ARGs-OAP Pipeline v2.0 (38).

To determine the alpha diversity (richness and evenness) within each of the ESBL-producing *E. coli*-originating wastewater samples, the Shannon diversity, richness, and evenness for each wastewater sample’s microbiome and resistome were calculated in R (version 4.2.2) Vegan package (39). The differences between treatment groups (i.e., sample types) were determined by calculating the beta diversities using the Bray-Curtis dissimilarity and Vegan package. Permutational multivariate analysis of variance (PERMANOVA) test Adonis in the Vegan package was used to determine the variance between treatment groups. The PERMANOVA test identifies statistical significance between treatment groups by reducing the dimensionality of the data. We used hierarchical cluster analysis to create heatmaps of the microbial genera (present above 0.1%) and ARGs across samples using the Vegan package. In the heatmaps, dendrograms were generated using Euclidean distances. To identify the associations between the co-occurrences of microbial communities and resistome, pairwise Spearman correlations were determined. Statistically correlated (*P* < 0.01) microbial genera and ARGs were visualized as a network with the R package igraph (40).

## RESULTS

### Distribution of ARGs in ESBL-producing *E. coli* genomes

**Fig 1:**
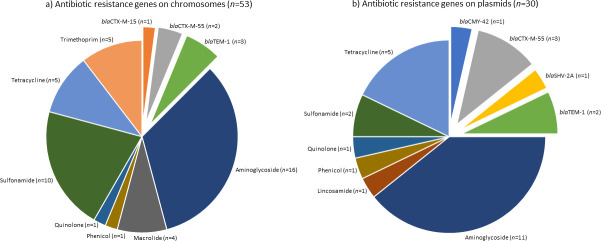
Distribution of antibiotic resistance genes on (**a**) chromosomes (*n* = 53) and (**b**) plasmids (*n* = 30) within the nine resolved *E. coli* genomes. The exploded slices in each pie demonstrate the extended-spectrum beta-lactam-associated genes. The numbers in parenthesis show the incidences of these genes across the nine resolved *E. coli* genomes.

We used the hybrid assembly of short- and long-read sequences to identify the location and distribution of beta-lactams and other ARGs on plasmids and chromosomes of wastewater-isolated ESBL *E. coli*. Nine of the 11 *E. coli* genomes were fully resolved with the circularization of chromosomes and plasmids. Two of the isolates, ESBL-A and ESBL-H, were not resolved. Of the nine resolved genomes, isolate ESBL-E had the longest chromosome, with a length of 5.04 Mbp, and isolate ESBL-I had the shortest chromosome with a length of 4.62 Mbp. In the 9 resolved genomes, 15 plasmids were identified and typed via pMLST (Table S4). Of the 15 plasmids, 6 carried ESBL-associated genes (pTEM1 in ESBL-B, Fig. S1; pCTXM55 in ESBL-C, Fig. S2; pSHV2A in ESBL-F, Fig. 3; pTEM1 in ESBL-G, Fig. S3; pCTXM55 plasmid in ESBL-I *E. coli* isolate in ESBL-I, Fig. S4; and pCTXM55 in ESBL-J, Fig. S5). There were 83 ARGs identified by AMRFinder across the nine resolved genomes, with 63.9% (*n* = 53) of these ARGs located on the chromosomes and 36.1% (*n* = 30) contained on plasmids of *E. coli* isolates ESBL-B, ESBL-C, ESBL-F, ESBL-G, ESBL-I, and ESBL-J (Fig. 1; Table S3).

Results demonstrate the presence of beta-lactams and other ARGs on plasmids and chromosomes of all but one resolved *E. coli* genomes (ESBL-E did not carry any ARGs; Table S3). Most *E. coli* genomes showed ARGs on both chromosomes and plasmids. Some *E. coli* isolates, however, carried ARGs only on their chromosomes (e.g., ESBL-D and ESBL-K) or plasmids (e.g., ESBL-F). Aminoglycoside resistance genes (*aac*3, *aad*A1, *aad*A5, *aad*A22, *aph*3, and *aph*6) were the most common ARGs on both plasmids and chromosomes, making up 30.2% (*n* = 16) and 36.7% (*n* = 11) of ARG presence, respectively (Fig. 1; Table S3). Beta-lactam genes were the second most abundant class of ARGs at 23.3% (*n* = 7) on plasmids (Fig. 1b) and at 11.3% (*n* = 6) on the chromosome (Fig. 1a). Five different beta-lactam genes were carried among the nine isolates: *bla*CTX-M-15, *bla*CTX-M-55, *bla*TEM-1, *bla*CMY-42, and *bla*SHV-2A. Of these beta-lactam genes, *bla*CTX-M and *bla*TEM types were the most common. *bla*CTX-M-15 was only seen on the chromosome of isolate ESBL-K. *blaCTX*-M-55 gene, however, was harbored by the chromosomes of ESBL-B and ESBL-D and plasmids of *E. coli* isolates ESBL-C, ESBL-I, and ESBL-J. *bla*TEM-1 was carried on plasmids of isolates ESBL-B and ESBL-G and chromosomes of ESBL-G, ESBL-I, and ESBL-J. The *bla*CMY-42 and *bla*SHV-2A genes were only detected in ESBL-G and ESBL-F, respectively, and were located on plasmids. Sulfonamide resistance genes (*sul*1, *sul*2) were relatively common on the chromosomes with six isolates carrying at least one, while only plasmids of isolates ESBL-F and ESBL-J had a *sul*1 and a s*ul*3 gene, respectively. The macrolide (*mph*A) and trimethoprim (*dfra*17) resistance genes were only chromosomally located in this isolate set. Tetracycline efflux transporters (*tet*A and *tet*B) were observed on five chromosomes and four plasmids. The prevalence of ARGs including beta-lactams on both chromosomes and plasmids of wastewater-originated *E. coli* isolates demonstrates the integration of these genes in the *E. coli* genomes (in chromosomes) and the path for their dissemination in the environment (via plasmids).

### Beta-lactams and other ARGs clustered on *E. coli* chromosomes

**Fig 2:**
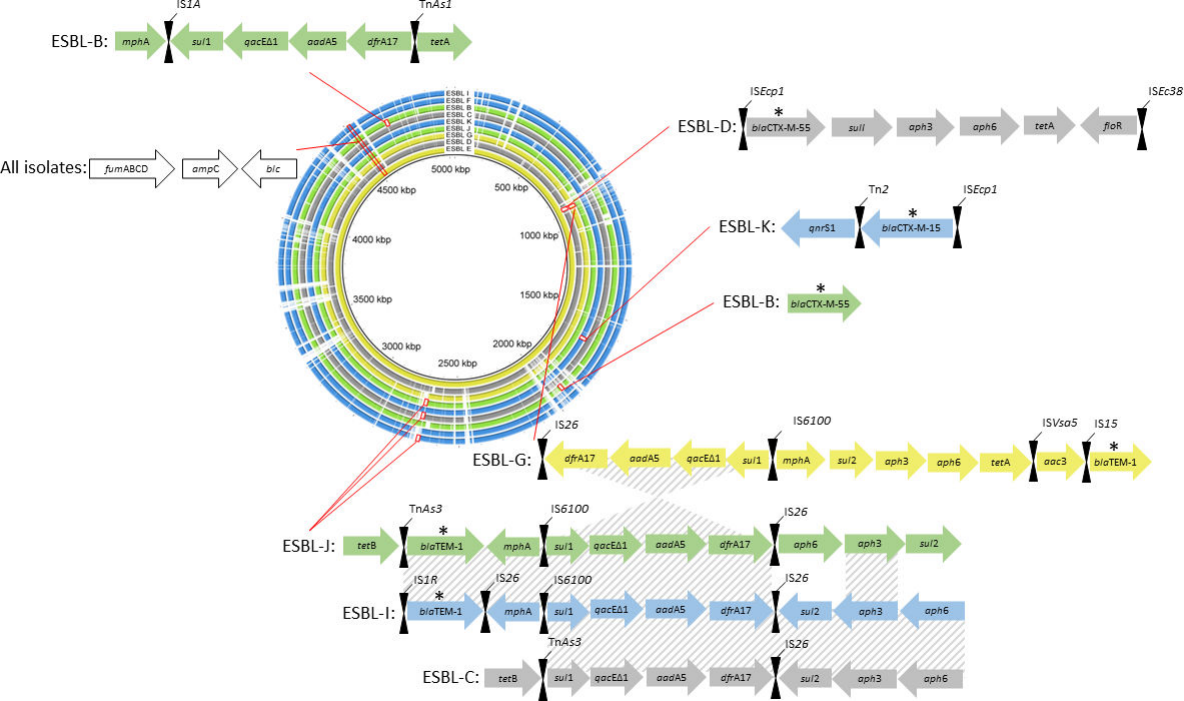
Alignment of the nine resolved ESBL)positive *E. coli* chromosomes and locations of their ARGs (arrows) and nearby transposases (hourglasses). Isolates are ordered by their chromosomes’ length from the longest in the innermost ring (isolate ESBL-E with a length of 5,036,705 bp) to the shortest in the outermost ring (isolate ESBL-I with a length of 4,626,801 bp) with the origin at the replication origin. Ring and annotation colors are according to the source sample type: green—influent, yellow—secondary effluent, blue—final effluent, and gray—biosolids. Areas of conserved synteny in the ARG regions are highlighted with diagonal stripes. Beta-lactam genes are indicated by an asterisk over the arrows.

We aligned the resolved ESBL *E. coli* chromosomes to determine the distribution of ARGs including beta-lactams and to identify the areas of conserved synteny (Fig. 2). Seven of the nine resolved *E. coli* chromosomes carried ARGs as detected by AMRFinder, including six chromosomes that carried either *bla*CTX-M or *bla*TEM type beta-lactams. BLAST alignment of the *E. coli* chromosomes shows over 90% nucleotide identity across the nine isolates, with three areas of dissimilarity where AMR genes were found in multiple isolates indicative of regions where accessory genes, such as ARGs, are located (Fig. 2). The majority of the annotated ARGs were clustered in these three variable regions, and interestingly, the ARGs were clustered together on their respective chromosomes with hits for transposon and insertion sequence-associated genes including Tn*2*, Tn*As1*, Tn*As3*, IS*1A*, IS*1R*, IS*15*, IS*26*, IS*6100*, IS*Ecp1*, IS*Ec38*, and IS*Vsa5*. The exception to the clustering of ARGs on these variable regions is the *bla*CTX-M-55 of Isolate ESBL-B, which was approximately 2 Mbp apart from an ARG cluster (i.e., *mph*A, *sul*1, *qac*E, *aad*A5, *dfra*A12, and *tet*A). In Isolates ESBL-C, ESBL-I, and ESBL-J, the chromosomal ARGs were all clustered in the same region between 2.67 and 2.68 Mbp (Fig. 2); all three of these isolates were identified as sequence type ST 744 (Table S1). Notably, these three isolates with the same sequence type carried a similar array of ARGs in the same region of the chromosome, with a *sul*1*-qac*E1*-aad*A5*-dfr*A17*-*IS*26* region in full alignment with each other (Fig. 2). Isolate ESBL-G shared this region, but on the opposite strand and at a distinct region of the chromosome. Isolates ESBL-J and ESBL-I carried a *bla*TEM-1 followed by macrolide resistance *mph*A on the opposite strand, while isolate ESBL-C did not have a beta-lactam gene in this region nor anywhere else on its chromosome. As expected, all nine resolved chromosomes carried the *amp*C encoding beta-lactamase, which was identical across all isolates and located in the identical region of the chromosome, at the end of a fumarase reductase operon (*fum*ABCD) and before a *blc* outer membrane lipoprotein (Fig. 2). These results demonstrate the genomic similarities between phenotypically confirmed ESBL isolates. These similarities include the location and presence of the *amp*C gene in all the genomes and a few identified areas of conserved synteny in the ARG regions suggestive of the integration of these regions in the *E. coli* genome.

### Conjugable plasmids and their resistance determinants

We conducted conjugation assays (with azide-resistant *E. coli* J53 as the recipient) to confirm the horizontal transfer of plasmid-mediated beta-lactam genes. Five of the nine isolates (ESBL-B, ESBL-C, ESBL-F, ESBL-G, and ESBL-J) produced transconjugants resistant to cefotaxime (100 µg/mL) and azide (5 µg/mL). These five isolates that produced transconjugants carried beta-lactam-associated genes on plasmids as observed in their resolved genomes (Table S3; extrachromosomal, circularized contigs). ESBL-I was the only isolate with a plasmid-borne beta-lactam gene that did not produce transconjugants in the assay. The AMR genotypes of six randomly selected transconjugant colonies were tested via PCR. Generally, ARGs co-located with the beta-lactam genes on the same plasmids were observed in the transconjugants, demonstrating the transfer of the plasmids to the recipient *E. coli* (Table S5). For isolate ESBL-C, *sul*1 and *aad*A5 were detected in the transconjugants, whereas these genes were not assembled on ESBL-C’s plasmid (Fig. S2). Closer analysis showed that *sul*1 and *aad*A5 in ESBL-C were co-located between two identical terminal repeat regions on the chromosome, which may have contributed to the misassembly of this region. The conjugation experiments demonstrate the widespread horizontal transfer of plasmid-mediated ARGs including beta-lactams. Accordingly, the prevalence of ARGs on plasmids can result in the pervasive dissemination of these genes in the environment.

**Fig 3:**
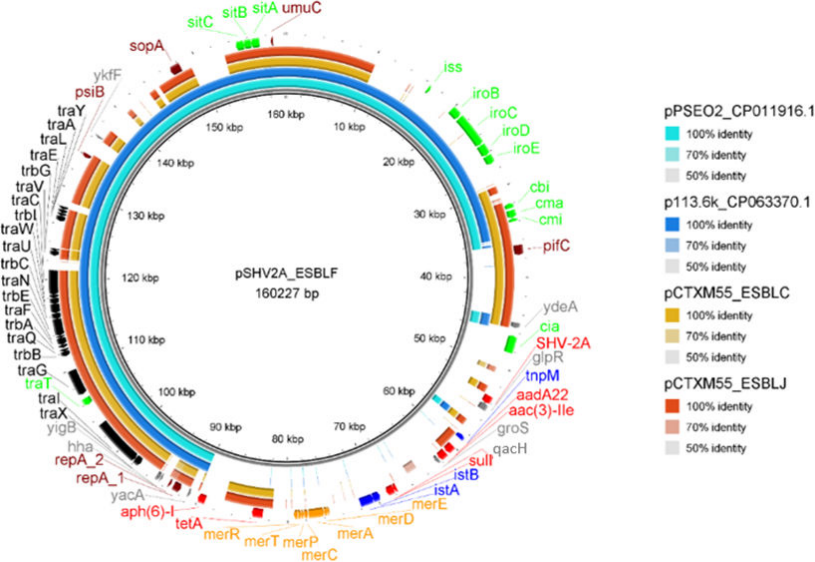
Alignment of pSHV2A plasmid in ESBL-F *E. coli* isolate and other similar beta-lactam-harboring IncF plasmids from this study as well as BLAST hits from the GenBank database. Outer two rings (dark- and light-orange) represent two similar IncF plasmids from this study (pCTXM55 plasmid in ESBL-J and pCTXM55 plasmid in ESBL-C *E. coli* isolates). Inner rings (dark- and light-blue) represent the closest matches to the pSHV2A plasmid in ESBL-F in the GenBank database. Gene labels are colored according to protein function: green—virulence factor, black—conjugation, maroon—plasmid replication/maintenance, red—antibiotic resistance gene, blue—mobile genetic element, and gold—mercury resistance.

Of the 15 plasmids identified in the resolved *E. coli* genomes, six carried beta-lactam genes (ESBL-B, ESBL-C, ESBL-F, ESBL-G, ESBL-I, and ESBL-J; Fig. 3; Fig. S1 to S5). These plasmids had some similarities and a few unique characteristics in terms of ARGs, virulence factors, and incompatibility groups compared with one another and with other plasmids previously deposited on GenBank. The beta-lactam-harboring plasmids of ESBL-C, ESBL-F, and ESBL-J were classified as IncF type, which has a narrow host range of Enterobacteriaceae, is common within *E. coli* genomes, and commonly harbors virulence factors and ARGs (pCTXM55_ESBLC, pSHV2A_ESBLF, and pCTXM55_ESBLJ in Fig. S2 and S5; Fig. 3, respectively; Table S4). Alignment analysis of these three IncF plasmids and the closest BLAST hits to pSHV2A_ESBLF (which was the most unique plasmid identified in our study) is shown in Fig. 3. All three plasmids (pCTXM55_ESBLC, pSHV2A_ESBLF, and pCTXM55_ESBLJ) contained a *tra* conjugation operon identical to two plasmids previously deposited on GenBank (p1 accession CP059932.1 and pMCR-PA accession CP29748.1), as well as *rep*A, *psi*B, *umu*C, and *sop*A genes related to plasmid maintenance/replication. All three IncF plasmids also carried the *cbi*, *cma*, and *cmi* colicin virulence factors. The 113-kbp long plasmid pCTXM55_ESBLC was identical to regions of two other plasmids observed in GenBank (p1 accession CP059932.1 and pRHB02-C06 accession CP058075.1) and contained seven virulence factors including the colicins, *iuc*ABCD, *hly*F, and *omp*T (Fig. S2).

pCTXM55_ESBLC had AMR genotypes of tetracycline (*tet*A) and ESBL (*bla*CTX-M-55). The 122-kbp plasmid pCTXM55_ESBLJ (Fig. S5) was nearly identical to the same GenBank plasmid as pCTXM55_ESBLC (p1 accession CP059932.1), except for three ~200-bp regions that contained IS*tB*, Tn*pB*, *and* Tn*pA26* transposases. pCTXM55_ESBLJ had a MDR genotype for beta-lactams (*bla*CTX-M-55), tetracyclines (*tet*A) and aminoglycosides (*aac*3, *aad*A1, *aad*A22), sulfonamides (*sul*3), and florfenicol (*flo*R). pSHV2A_ESBLF was the most unique plasmid observed in our study (Fig. 3). This plasmid shared the conjugation and partitioning machinery of the other two IncF plasmids (pCTXM55_ESBLC and pCTXM55_ESBLJ) but was unique in the variable region (Fig. 3, 45 kbp–90 kbp) that stored the plasmid’s ARGs. This region also carried a *mer* mercury resistance operon. pSHV2A_ESBLF contained ARGs for four antibiotic classes: beta-lactams (*bla*SHV-2A), aminoglycosides (*aad*A22, *aac*3, and *aph*6), tetracyclines (*tet*A), and sulfonamides (*sul*1). The conserved conjugation and replication region was identical to two plasmids within GenBank: p113.6k (accession CP063370.1) and pPSEO2 (accession CP011916.1). Therefore, while we observed the similarities of the pSHV2A_ESBLF in the conjugation and partitioning machinery regions with other plasmids in our study and on GenBank, this plasmid harbored a unique region carrying multiple ARFs as well as a mercury resistance operon. Such unique regions on plasmids can easily spread via horizontal gene transfer to other hosts within the environment spreading ARGs and virulence factors.

Three other *E. coli* genomes harbored beta-lactam genes and were classified as IncN and IncI compatibility types (ESBL-B, ESBL-G, and ESBL-I in Fig. S1, S3 and S4, respectively). Isolates ESBL-B and ESBL-G carried plasmids with *bla*TEM-1 genes of the IncN and IncI incompatibility types, respectively. pTEM1_ESBLB, the smallest ESBL-harboring plasmid observed in these isolates (43.8 kbp), had a *pil*X operon related to conjugable pili generation and a *res-par*GF plasmid partitioning system. The *bla*TEM-1 ESBL gene was the only ARG on this plasmid, with similarity to the plasmid pOLA52 (accession EU370913.1). pTEM1_ESBLG, a 69.7-kbp IncI-type plasmid, harbored a *bla*TEM-1 and *bla*CMY-42 beta-lactamase along with a *qnr*S1 quinolone resistance gene. Interestingly, *bla*CMY-42 was flanked by a *blc* lipoprotein, similar to the *amp*C gene detected in all nine resolved chromosomes. No virulence factors in the VirFinder database were detected on this plasmid. The area containing ARGs on pTEM1_ESBLG was not detected on any plasmids in GenBank, while the area containing the conjugation genes *tra*A*-trb*AB*-nik*AB matched those of the previously described plasmid pS68 (accession KU130396.1). The plasmid assembled from isolate ESBL-I was not observed to transfer via conjugation but carried *bla*CTX-M-55, *tet*A, and *aac*3 ARGs, aerobactin synthetase virulence factors (*iuc*BCD), and IncI-type alleles. While the similarities observed in the plasmids of this study with others on GenBank demonstrate the widespread dissemination of these plasmids in the environment, the unique regions suggest mutations within the genomes. These mutated regions that carry ARGs and virulence factors can disseminate via horizontal gene transfer to other hosts contributing to the emergence of pathogenic multidrug resistance genotypes.

### Virulence factors co-located with beta-lactams

We identified 115 virulence factors on the resolved *E. coli* genomes (Table 1). Sixty-seven percent (*n* = 77) of the virulence factors was located on chromosomes, with all nine chromosomes carrying at least two virulence factors. The other 38 virulence factors were located on plasmids that also harbored beta-lactam-encoding genes (Table 1). The colicin virulence factors (*cba*, *cia*, *cib*, and *cma*) were notable with at least one observed on all the six resolved plasmids that carried beta-lactam genes. Aerobactin synthetase and receptor genes *iuc*C and *iut*A were observed on the three plasmids that carried *bla*CTX-M-15. Colicin virulence factors, *iuc*C and *iut*A, were all unique to plasmids, with no occurrences on chromosomes. The virulence-related iron transport protein *sit*A was found in three chromosomes (ESBL-B, ESBL-E, and ESBL-K) and in four plasmids (ESBL-C, ESBL-F, ESBL-I, and ESBL-K). The most common virulence factors in their genomes, glutamate decarboxylase (*gad*) and tellurium ion resistance protein *ter*C, were chromosomal only, with multiple isolates having redundant copies of these genes. The presence of virulence factors on bacterial genomes is critical to understanding bacterial pathogenicity. The co-location of virulence factors and ARGs suggests resistance to the treatment of bacterial infections. Moreover, the co-occurrences of virulence factors and ARGs on the plasmids contribute to the emergence of these potentially pathogenic antibiotic-resistant bacteria in environmental reservoirs.

**TABLE 1:** Distribution of virulence factors on chromosomes (C) and plasmids (P) in ESBL-producing *E. coli* isolates

Virulence factor function	Gene(s)	ESBL *E. coli* isolate ID								
		B	C	D	E	F	G	I	J	K
Aerobactin synthetase	*iuc*C		P^*a*^		–			P^*a*^	P^*a*^	
Afimbrial adhesion	*afa*D						C			
Capsule polysaccharide export	*kps*E			C	C					
Colicin	*cba, ci(ab), cma*		P^*b*^		P^*b*^	P^*b*^		P^*b*^	P^*b*^	P^*b*^
Enterobactin siderophore receptor protein	*iro*N					P^*c*^				
Ferric aerobactin receptor	*iut*A		P^*a*^					P^*a*^	P^*a*^	
Glutamate decarboxylase	*gad*	C	C	C	C	C	C	C	C	C
Heat-resistant agglutinin	*hra*			C					C	
Hemolysin F	*hly*F		P^*a*^			P^*c*^		P^*a*^	P^*a*^	
High molecular weight protein 2 non-ribosomal peptide synthetase	*irp*2				C					
Increased serum survival	*iss*	C		C		C, P^*c*^				
Iron transport protein	*sit*A	C	P^*a*^		C	P^*c*^		P^*a*^	P^*a*^	C
Long polar fimbriae	*ipf*A			C			D			D
Major pilin subunit F11	*pap*A_F11								C	
Major pilin subunit F19	*pap*A_F19			C						
Microcin	*cva*C*, mcm*A		P^*a*^			P^*c*^		P^*a*^	C	
Outer membrane hemin receptor	*chu*A			C	C					
Outer membrane protease (protein protease 7)	*omp*T		P^*a*^		C	P^*c*^		P^*a*^	P^*a*^	
Outer membrane protein complement resistance	*tra*T		P^*a*^			P^*c*^		P^*a*^	P^*a*^	
Outer membrane usher	*pap*C*, afa*C		C						C	
Periplasmic chaperone	*afa*B								C	
Polysialic acid transport protein; group 2 capsule	*kps*MII_K5		C							
Siderophore receptor	*fyu*A*, ire*A				C				C	
Tellurium ion resistance protein	*ter*C	C	C		C	C	C	C	C	C
Transcriptional regulator	*afa*A								C	
EAST-1 heat-stable toxin	*ast*A		C							
Enteroaggregative immunoglobulin repeat protein	*air*		C		C					
*Salmonella* HilA homolog	*eil*A		C		C					

^*a*^ On *bla*CTX-carrying plasmid.

^*b*^ On all beta lactam-carrying plasmids.

^*c*^ On *bla*SHV-carrying plasmid.

### Metagenomics

The alpha diversity indices (i.e., Shannon diversity, richness, and evenness) of the microbial genera and ARGs for the 11 wastewater samples are provided in Table S6. Alpha diversity indices can provide critical insights into the structure and function of microbial communities and their resistome. The alpha diversity of the resistome helps in understanding the capacity of microbial communities to withstand antibiotic treatments and can indicate the potential for resistance to spread. The richness of microbial genera, which demonstrates the total number of microbial genera, ranged from 5,000 to 5,500 except for final effluent sample I, which was an outlier with a richness of 3,220. This sample’s microbial composition had a Shannon index of 4.96, similar to those of the three influent samples A, B, and J (4.91, 4.77, and 4.93, respectively). All other samples had Shannon indices of 6.0 to 6.3 in the genera data set. The resistomes of influent samples A, B, and J had a richness that ranged between 355 and 378 unique ARGs, while the remainder of the samples were less rich, with 144 to 277 unique ARGs. Shannon diversity indices for ARG abundance ranged from 3.09 to 4.48 in all samples, indicating that all the eleven wastewater samples had similar diversity of unique ARGs. Overall, the 11 samples had similar alpha diversity indices except for sample I, which had a lower richness of microbial genera. Diverse microbial communities in wastewater treatment systems are indicators of ecosystem health and stability and can enhance the degradation of a wide range of pollutants. Monitoring the diversity of microbial communities and resistome in wastewater helps in assessing the public health risks associated with the environmental impact of the treated effluent and biosolids on the receiving environments.

Bray-Curtis dissimilarity scores (herein referred to as dissimilarity), non-metric multidimensional scaling (NMDS) plots, and PERMANOVA were used to determine the differences within and between the ESBL-producing *E. coli*-originating wastewater samples with respect to microbial community and resistome (Fig. S6, S7a and b). PERMANOVA analysis identified that for both the microbial community and the resistome, the dissimilarities between each treatment group (i.e., sample type) were statistically higher than the differences within each treatment group (microbial community: *P* < 0.001; resistome: *P* < 0.01). Biosolids samples (C, D, and H) were similar to each other with respect to their microbial community composition (dissimilarity = 0.196) and, to a lesser degree, their resistome (dissimilarity = 0.363). The same applied to secondary effluent samples (E, G) (dissimilarity = 0.288 for genera, 0.363 for ARGs). Influent samples (A, B, and J) were equally similar with regard to both data sets (dissimilarity = 0.276 for genera, 0.222 for ARGs). Effluent samples (F, I, and K) were less similar to one another with dissimilarity scores greater than 0.5 in both data sets. These findings demonstrate the similarity within and the dissimilarities between wastewater sample types (i.e., influent, secondary effluent, final effluent, and biosolids) in terms of microbial communities and resistome. While these wastewater samples were collected from eight utilities (Table S1), they have similar microbial communities and resistome.

**Fig 4:**
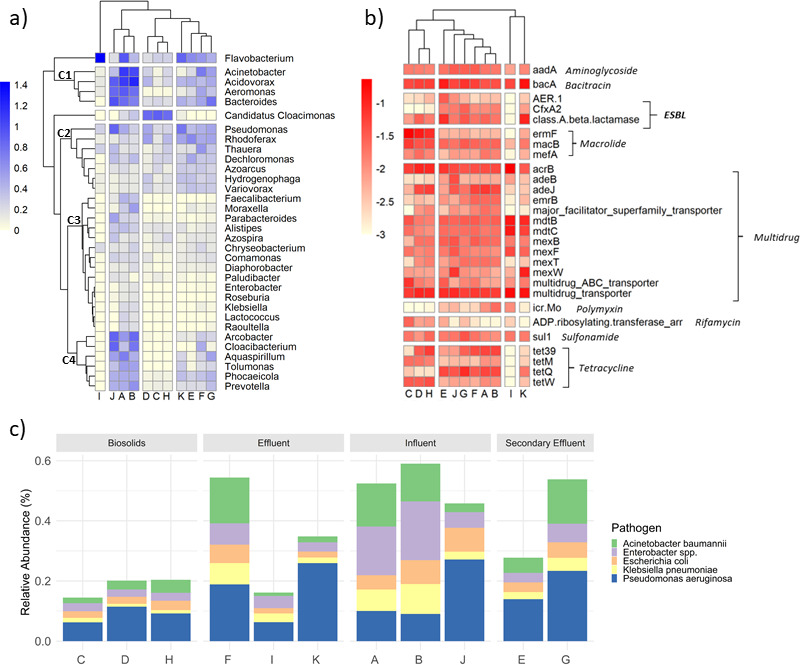
Microbial community and resistome of ESBL-producing *E. coli*-originating wastewater samples. Letters A–K correspond to wastewater sample IDs. (**a**) Heatmap of the relative abundance of the microbial genera present above 0.1% (log-scale). (**b**) Heatmap of the antibiotic resistance gene concentrations (gene copies/16S rRNA, log-scale) grouped by their corresponding antibiotic class. (**c**) Relative abundance of ESKAPE pathogens and *E. coli* with samples grouped according to wastewater sample type.

The most abundant microbial genera (>0.1% relative abundance) are displayed in the heatmap in Fig. 4a. These results describe the dominant microbial genera and identify the differences and similarities observed between the ESBL-producing *E. coli*-originating wastewater samples in the microbial genera NMDS plot (Fig. S7a). Biosolids samples (D, C, and H) included *Candidatus cloacimonas* as their most abundant genus. The high abundance of *Candidatus cloacimonas* and the lower concentration of other genera as compared with wastewater influent, secondary effluent, and effluent contribute to the clustering of biosolids samples together (toward the top on Fig. S7a) and apart from other clusters in the NMDS plot in Fig. S7a (i.e., driving the separation on NMDS2 axis). Influent samples (A, B, and J) had two dominant clusters C1 and C4 highlighted in Fig. 4a. Cluster C1 contains *Acinetobacter*, *Acidovorax*, *Arcobacter*, *Aeromonas*, and *Bacteroides*, and cluster C4 includes *Arcobacter*, *Cloacibacterium*, *Aquaspirillum*, *Tolumonas*, *Phocaeicola*, and *Prevotella* (Fig. 4A). Majority of these 11 genera belong to *Proteobacteria* phylum, and a few are *Campylobacterota* and *Bacteroidota*. The two clusters C1 and C4 could be the driving factors for the separation of influent samples from other sample types to the left of the NMDS1 axis on Fig S7a. The final effluent sample I harbored dissimilar genera compared with the other two effluent samples F and K (Fig. 4a). Sample I has a higher abundance of *Flavobacterium* and lower abundances of clusters C2 and C4 compared with samples F and K. This dissimilarity can explain the separation of sample I toward the right on NMDS1 axis of Fig. S7. These findings demonstrate that the differences between the treatment groups (i.e., sample types and microbial community: *P* < 0.001 PERMANOVA) can be described by the differences in the abundances of clusters C1, C2, C4, and *Flavobacterium*.

The dominant ARGs (>0.02 gene copies/16S rRNA) are shown in Fig. 4b. Findings illustrate the abundant ARGs in different wastewater sample types and can describe the similarities between and within the samples in clusters of NMDS plot Fig. S7b. The higher dissimilarity of biosolids samples C, D, and H from other sample types (Fig. S7b) can be related to a few ARGs: the high abundance of macrolide resistance *erm*F and tetracycline resistance *tet*W biosolids samples, while not as common in other sample types, and tetracycline resistance *tet*Q is at lower levels in biosolids as compared with the abundance of this gene in other sample types. The bacitracin *bac*A, aminoglycoside *aad*A, sulfonamide *sul*1, and multidrug *acr*B were common ARGs in all 11 samples. The most prevalent beta-lactam genes in these wastewater samples were Type A beta-lactamases, which is the class that includes *bla*SHV, *bla*TEM, and *bla*CTX-M type beta-lactams. These were the most abundant in the secondary effluent sample E and the final effluent sample K. The beta-lactam genes *bla*AER.1 and *bla*CfxA2 were also detected in the same samples as class A beta-lactamases. These findings suggest *erm*F, *tet*W, and *tet*Q as the main ARGs that cause dissimilarity between biosolids and other sample types and the separation on NMDS1 axis of Fig. S7b.

To determine the prevalence and abundance of ESKAPE (six highly virulent and antibiotic-resistant bacterial pathogens including *Enterococcus faecium*, *Staphylococcus aureus*, *Klebsiella pneumoniae*, *Acinetobacter baumannii*, *Pseudomonas aeruginosa*, and *Enterobacter* spp., as well as *E. coli*; Fig. 4c) in different sample types, species abundance data were parsed for these target pathogens. The most abundant ESKAPE pathogen species in all sample types was *P. aeruginosa*, which ranged from 0.6% to 2.7% of the microbial community (Fig. 4c). *S. aureus* and *E. faecium* were below 0.01% abundant in all 11 samples. *E. coli* was less than 0.1% of the microbial community in all samples. Our results identify the prevalence of four of the six ESKAPE pathogens as well as *E. coli* in the 11 wastewater samples including—from higher to lower abundance—*P. aeruginosa*, *A. baumannii*, *Enterobacter* spp., and *K. pneumoniae*.

**Fig 5:**
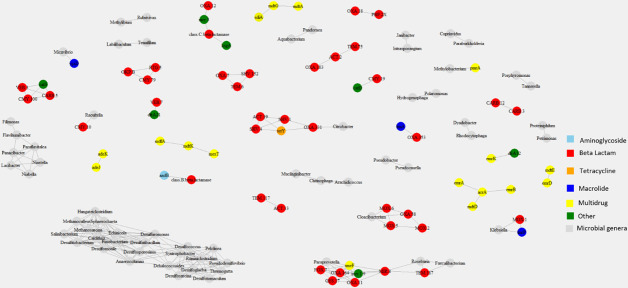
Network analysis of microbial genera and antibiotic resistance genes in wastewater samples. Edges represent statistically significant Spearman correlations (*P* < 0.01) of nodes with a neighborhood of three or more. Colored symbols indicate antibiotic resistance genotypes, and gray symbols represent the microbial genera.

Analysis of the prevalence and abundance of ARGs including beta-lactam genes between the *E. coli* isolates and their originating wastewater samples revealed that of the ARGs found in *E. coli* isolates, the most abundant in the wastewater samples were *sul*1 (0.004–0.023 gene copies/16S rRNA), *qac*EΔ1 (0.001–0.020 gene copies/16S rRNA), *aad*A (0.002–0.014 gene copies/16S rRNA), *sul*2 (undetected–0.007 gene copies/16S rRNA), and *tet*A (0.001–0.005 gene copies/16S rRNA) (Table S7). The beta-lactam genes *bla*CTX-M-55 and *bla*TEM-1, which were identified in *E. coli* isolates, were detected in one (sample B) and five wastewater samples (samples B, E, F, J, and K) from five unique facilities, respectively, at levels greater than 0.001 gene copies/16S rRNA. Beta-lactam genes *bla*CTX-M-15, *bla*SHV-2A, and *bla*CMY-42, also identified in *E. coli* isolates, were undetected in all source samples. Seven beta-lactam ARGs were detected above 0.001 gene copies/16S rRNA in at least three samples (Table S8): class A beta-lactamases (<0.001–0.061 gene copies/16S rRNA), *bla*AER-1 (<0.001–0.013 gene copies/16S rRNA), *bla*CfxA2 (<0.001–0.011 gene copies/16S rRNA), *bla*OXA-2 (<0.001–0.009 gene copies/16S rRNA), *bla*OXA-10 (<0.001–0.007 gene copies/16S rRNA), *bla*CfxA3 (<0.001–0.002 gene copies/16S rRNA), and *bla*OXA-119 (<0.001–0.002 gene copies/16S rRNA). Moreover, correlation analysis between the ARGs and microbial genera in wastewater samples demonstrates statistical associations between the microbial genera and 39 beta-lactam-associated genes (Fig. 5). None of the specific beta-lactam genes found in the *E. coli* isolates were statistically correlated (*P* < 0.01) with the microbial genera in ESBL-producing *E. coli*-originated wastewater samples. The prevalence and abundance of *Cloacibacterium* genera, which was only detected above 0.01% abundance in all three influent samples (A, B, and J) and one final effluent sample (F), correlated with *bla*OXA.58 and *bla*MOX.2,5,6 beta-lactamases (Spearman’s ρ = 0.989, *P* < 0.01). *Paraprevotella* correlated with *bla*OXA.11, *bla*FOX.7, *bla*GES.17, and *bla*OXA.164 (Spearman’s ρ = 0.985, *P* < 0.01), along with the colistin resistance gene *mcr.1.9* (Spearman’s ρ = 0.985, *P* < 0.01) and the multidrug efflux pump *sme*F (Spearman’s ρ = 0.985, *P* < 0.01). These were in the same cluster as the genera *Faecalibacterium* and *Roseburia* with the beta-lactam-associated gene *bla*TEM.187. Another colistin resistance gene *mcr.3* correlated with the class C beta-lactamase class (Spearman’s ρ = 0.986, *P* < 0.01), including *amp*C enzymes. The ESKAPE pathogen genus *Klebsiella* was correlated with *bla*MOX.1 (Spearman’s ρ = 1.000, *P* < 0.01) and the multidrug ARG *lmr*P (Spearman’s ρ = 1.000, *P* < 0.01). The beta-lactam-related genes *bla*SHV.1 and *bla*SHV.4 co-occurred with the tetracycline resistance gene *tet*Y (Spearman’s ρ = 0.985, *P* < 0.01) in final effluent, secondary effluent, and influent samples. The beta-lactams *bla*TEM.6, *bla*SHV.152, and *bla*OXA.7 were also statistically correlated with each other (Spearman’s ρ = 0.985, *P* < 0.01). Overall, we identified seven ARGs that were present in both *E. coli* genomes and their originating wastewater samples including *sul*1, *qac*EΔ1, *aad*A, *sul*2, *tet*A, and beta-lactams *bla*CTX-M-55 and *bla*TEM-1. Moreover, we found statistically significant associations between the microbial genera and ARGs including beta-lactams in wastewater samples including co-occurrences of *Klebsiella* ESKAPE pathogen with beta-lactam *bla*MOX.1 and the multidrug ARG *lmr*P. These results are important in understanding the potential risks of ARGs including the clinically important beta-lactams and their dissemination in bacterial pathogens in wastewater systems.

## DISCUSSION

Using a hybrid sequencing and assembly strategy (short- and long-read sequencing), we fully resolved the genomes of nine wastewater-originated *E. coli* isolates with the circularization of chromosomes and plasmids. Our method allowed us to identify the locations and orientations of ARGs and virulence factors harbored on plasmids and chromosomes of the nine ESBL-producing *E. coli* isolates. We detected class A beta-lactamase genes in several of the ESBL-producing *E. coli* and their originating wastewater samples. Worldwide, class A beta-lactamases, such as those derived from *bla*TEM-, *bla*CTX-, and *bla*SHV-type genes, are the most detected in Enterobacterales in the agricultural supply chain (41). These ESBL-producing *E. coli* isolates carried ARGs associated with nine antibiotic classes distributed on both chromosomes and plasmids. On chromosomes, ARGs were co-located together within variable regions in close proximity to various transposases or insertion elements (Fig. 2). This co-localization of mobile genetic elements and ARGs suggests that transposition events were originally responsible for the accumulation of these ARGs in the genome. Moreover, there was conserved synteny between the ARGs of three isolates chromosomes (ESBL-C, ESBL-I, and ESBL-J, collected from three different samples from two different utilities; Table S1) belonging to sequence type ST 744 at the *sul*1*-qac*E1*-aad*A5*-dfr*A17*-*IS26 region, indicative of a similar origin for these ARGs in these three *E. coli* isolates. All nine *E. coli* isolates in this study were phenotypic ESBL producers and carried a chromosomal *amp*C beta-lactamase that was homologous across the chromosomes. In combination with beta-lactam genes like *bla*TEM-1 and *bla*CTX-M-55, overexpression of these chromosomal *amp*C genes can have an additive effect on their beta-lactam resistance phenotype (42).

The detection of beta-lactam-associated genes on conjugable plasmids indicates a potential for these genes to spread horizontally in wastewater treatment utilities and downstream ecosystems (Table S3). The identification of transposases nearby ARGs on plasmids reflects the chromosomal ARG organization, for example, pCTX_ESBLJ carried an IS*26*-related element (Tn*pA26*) within a few hundred base pair of aminoglycoside, sulfonamide, and tetracycline ARGs (Fig. S5). This may be indicative of the intercellular transposition of ARGs between chromosomes and plasmids. Future studies may explore how different environmental conditions may affect the persistence of ARGs on chromosomes after transposition from plasmids. Five of the isolates were observed to transfer plasmid-mediated ARGs between *E. coli* strains (Table S5). Three of these plasmids belonged to the IncF incompatibility group (Fig. S2 and S5; Fig. 3). These have been described as narrow-host range and may only transfer to other Enterobacteriaceae or remain stable within *E. coli* genomes (9). A unique plasmid pSHV2A_ESBLF was resolved, which carried a variable region with five classes of ARGs, a metal resistance operon (*mer*), and *iro*BCD and *sit*ABC virulence factors (Fig. 3). The co-localization of MDR determinants and metal resistance genes is concerning given the threat of antimicrobial-resistant pathogens in natural and built environments (43).

Conjugation assays give valuable insight into the transferability of plasmids containing ARGs. The behavior of these organisms at the laboratory scale, however, is likely not representative of the extent to which horizontal gene transfer occurs within wastewater treatment systems. Conjugation of plasmid pCTXM55_ESBLI was not observed with the experimental conditions used in this study. Many factors can influence conjugation at the laboratory scale, including incubation time, temperature, and donor and/or recipient concentrations (24, 44). The observation, or lack thereof, of conjugation in controlled experiments with only two *E. coli* strains does not have meaningful implications on the likelihood of the plasmid to propagate via HGT in wastewater treatment systems. The narrow-host range plasmids that carry these beta-lactam resistance genes may lack suitable hosts in the wastewater treatment ecosystem, as our metagenomic analysis showed a low (<0.1%) abundance of Enterobacterales. It should be noted that the conjugation assay used in this article selects for specific transconjugants, with cefotaxime and azide phenotypes. The selective pressure to isolate these transconjugants may not exist in a wastewater system. Furthermore, the transfer of beta-lactam genes is not limited to plasmids; the ability for ARGs to be relocated from chromosomes to conjugatable plasmids through transposition is a contributing factor to the mobilization of ARGs (45).

Metagenomics showed that multidrug efflux pumps, bacitracin resistance genes, and class A beta-lactamases were among the most common ARGs across the 11 wastewater samples from which the ESBL-producing *E. coli* were isolated (Fig. 4b). Community analysis of the wastewater samples showed *Acinetobacter*, *Acidovorax*, *Aeromonas*, and *Bacteroides* as the core genera in influent, secondary effluent, and final effluent (Fig. 4a). *Candidatus cloacimonas* was a dominant genus in the three biosolids samples, all of which were from separate wastewater treatment utilities (Fig. 4a). A correlation analysis showed that the beta-lactam resistance genes *bla*TEM.8, *bla*SHV.152, and *bla*OXA.7 co-occurred at similar rates within wastewater samples. The ARGs *sul*1 and *aad*A occurred in both wastewater samples as well as the *E. coli* isolates. The presence of *E. coli* was low (<0.1%) in these wastewater samples, but the location of these genes on conjugable plasmids and on IS*26* insertional elements in the *E. coli* genomes may explain the prevalence of these sulfonamide and aminoglycoside resistance genes within the samples. While class A beta-lactamases, in general, were abundant (>0.01 gene copies/16S rRNA), the specific beta-lactam genes *bla*CTX-M-55, *bla*CTX-M-15, *bla*TEM-1, *bla*SHV-2A, and *bla*CMY-42 that occurred in the *E. coli* isolates were below the detection limit in the ESBL-producing *E. coli*-originating wastewater samples.

### Conclusion

Here, we provide the genetic context of ESBL-producing *E. coli* isolated from wastewater samples. The synteny of the chromosomal genetic regions where ARGs were located and the presence of transposon and insertion sequence-associated genes in these regions are indicative of similar HGT origins of the resistance determinants. Having collected these isolates from various regions across the State of Oregon and at different stages of wastewater treatment systems, the primary concern is that these genes are likely to further proliferate in the natural environment.

The occurrence of plasmids associated with MDR and virulence genotypes, including a novel plasmid that also carried a mercury resistance operon, is significant given the role conjugation plays in the spread of AMR and virulence in the environment. However, the ESBL-harboring plasmids in the *E. coli* genomes were all characterized as narrow-host range, so it is possible that these genes may not readily spread to pathogens other than *E. coli* and close relatives. Additionally, the specific beta-lactam genes *bla*CTX-M-55, *bla*CTX-M-15, *bla*TEM-1, *bla*SHV-2A, and *bla*CMY-42 that occurred in the *E. coli* isolates were in low abundance (<0.001 gene copies/16S rRNA) in the *E. coli*-originating wastewater samples. This may be associated with the low relative abundance of Enterobacterales in wastewater samples. The cross-genera spread of MDR plasmids would be valuable for future research efforts.

The detection of a broad spectrum of ARGs and ESBL-producing *E. coli* isolates in biosolids and final effluents is notable given that these streams are transported to downstream ecosystems like rivers and agricultural fields. Through the use of hybrid whole-genome sequencing, a complete genetic context for these ESBL-producing *E. coli* was established. This approach provided insights into the potential HGT origins of the ARGs. The conjugation of ARG-harboring plasmids is a major mechanism for the proliferation of beta-lactam and other AMR determinants among *E. coli*, supported by five of the *E. coli* isolates producing cefotaxime-resistant transconjugants in the conjugation assays. Furthermore, metagenomic analysis of the source wastewater samples showed the presence of a variety of beta-lactam resistance genes in the wastewater ecosystem. Correlation analysis indicated some association of beta-lactam resistance genes and microbial genera such as *Cloacibacteria*, *Paraprevotella*, *Faecalibacterium*, and *Roseburia* found in the wastewater samples. Future studies may explore these associations in more depth, similar to what was done with the *E. coli* isolates in this study. The incorporation of whole-genomic, metagenomic, and culture-based methods provides a holistic picture of the ESBL-producing *E. coli* in wastewater systems and the broader community.

## Data Availability

The sequenced data sets have been deposited in NCBI: the *E. coli* genomes are available in GenBank under BioProject accession number PRJNA1044148 and the wastewater metagenomes are available in Short Read Archive (SRA) under BioProject accession number PRJNA1060321.

## SUPPLEMENTAL MATERIAL

Supplemental figures and tables spectrum.00717-24-s0001.docx Supplemental figures and tables

ASM does not own the copyrights to Supplemental Material that may be linked to, or accessed through, an article. The authors have granted ASM a non-exclusive, world-wide license to publish the Supplemental Material files. Please contact the corresponding author directly for reuse.
