# Supplementary material for: Resolved genomes of wastewater ESBL-producing Escherichia coli and metagenomic analysis of source wastewater samples
Source: Microbiol Spectr. 2024 Aug 21;12(10):e00717-24. doi: 10.1128/spectrum.00717-24 (PMC11448238; doi:10.1128/spectrum.00717-24)
Supplement: Supplemental figures and tables [file spectrum.00717-24-s0001.docx]

**Supplementary Information**

**Table S1.** Summary of ESBL-positive E. coli isolates (1). MLST: Multi-locus sequence type of E. coli. Phenotypes are listed if the isolate was resistant according to VITEK2 measurement (AMP: Ampicillin, CTX: Cefotaxime, CEF: Cefepime, TET: Tetracycline, SXT: Trimethoprim/Sulfamethoxazole, CPFX: Ciprofloxacin, AMC: Amoxicillin/Clavulanic Acid, GEN: Gentamicin).

| Isolate ID | Source ID | Utility ID | MLST | Antibiotic resistance phenotype |
| --- | --- | --- | --- | --- |
| ESBL-A | Influent (A) | 1 | 328 | AMP, CTX, CEF, TET, SXT |
| ESBL-B | Influent (B) | 1 | 46 | AMP, CTX, TET, SXT |
| ESBL-C | Biosolids (C) | 2 | 744 | AMP, CTX, CEF, CPFX, TET, SXT |
| ESBL-D | Biosolids (D) | 3 | 69 | AMP, CTX, CEF, TET |
| ESBL-E | Secondary Effluent (E) | 3 | 70 | AMP, CTX, TET, SXT |
| ESBL-F | Final Effluent (F) | 4 | 34 | AMP, CTX, TET |
| ESBL-G | Secondary Effluent (G) | 5 | 224 | AMP, AMC, CTX, GEN, CPFX, TET, SXT |
| ESBL-H | Biosolids (H) | 6 | 744 | AMP, CTX, CEF, GEN, CPFX, TET, SXT |
| ESBL-I | Final Effluent (I) | 7 | 744 | AMP, CTX, GEN, CPFX, TET, SXT |
| ESBL-J | Influent (J) | 7 | 744 | AMP, CTX, CEF, GEN, CPFX, TET, SXT |
| ESBL-K | Final Effluent (K) | 8 | 3580 | AMP, CTX, CEF |

**Table S2.** PCR primers and conditions

| Gene | Primers | Amplicon length | Annealing temperature | Reference |
| --- | --- | --- | --- | --- |
| *bla*_CTX-M_ | F: GGTTAAAAAATCACTGCGTC | 103 | 58°C | This Study^1^ |
|  | R: TTACAAACCGTCGGTGACGA |  |  |  |
| *bla*_TEM_ | F: AGATCAGTTGGGTGCACGAG | 403 | 60°C | This Study |
|  | R: TTCATTCAGCTCCGGTTCCC |  |  |  |
| *bla*_SHV_ | F: TCGCCTGTGTATTATCTCCC | 768 | 52°C | (2) |
|  | R: CGCAGATAAATCACCACAATG |  |  |  |
| *bla*_CMY_ | F: TGGCCAGAACTGACAGGCAAA | 462 | 62°C | (2) |
|  | R: TTTCTCCTGAACGTGGCTGGC |  |  |  |
| *aad* | F: CACTGGACACAATCCACCTG | 218 | 58°C | (3) |
|  | R: CCAAGGCACTACTTCGCTTC |  |  |  |
| *aph* | F: TTCATTGCCAGACGGGACTC | 485 | 60°C | This Study |
|  | R: CGACGGGATCTATCACCAGC |  |  |  |
| *mph* | F: TCGTTGCCTATCCCATGCTC | 334 | 59°C | This Study |
|  | R: TAGAGATCGCCATGCACCAC |  |  |  |
| *sul1* | F: TTCGGCATTCTGAATCTCAC | 822 | 59°C | (2) |
|  | R: ATGATCTAACCCTCGGTCTC |  |  |  |
| *tetA* | F: GGCCTCAATTTCCTGACG | 372 | 57°C | (4) |
|  | R: AAGCAGGATGTAGCCTGTGC |  |  |  |
| *dfrA* | F: CGCCTGGTTTAAACGCAACA | 151 | 60°C | This Study |
|  | R: CACCGACTTCACCCACGTTA |  |  |  |

^1^ Primers with reference to “This Study” refer to primers built with the NCBI Primer Design Tool using reference sequences from the accessions associated with AMRFinder hits for each gene.

**Table S3.** Distribution of ARGs on chromosomes (C) and plasmids (P) within the nine resolved ESBL *E. coli* genomes.

|  |  | ESBL isolate ID | | | | | | | | |
| --- | --- | --- | --- | --- | --- | --- | --- | --- | --- | --- |
| Antibiotic class | Gene(s) | B | C | D | E | F | G | I | J | K |
| Aminoglycoside | *aac*3-IId |  |  |  |  |  | C |  |  |  |
|  | *aac*3-IIe |  |  |  |  | P^1^ |  | P^2^ | P^3^ |  |
|  | *aac*3-VIa |  |  |  |  |  |  |  |  |  |
|  | *aad*A1 |  |  |  |  |  |  |  | P^3^ |  |
|  | *aad*A22 |  |  |  |  | P^1^ |  |  | P^3^ |  |
|  | *aad*A5 | C | C |  |  |  | C | C | C |  |
|  | *aph*3'-Ia |  | P |  |  |  |  | P | P |  |
|  | *aph*3''-Ib |  | C | C |  | P | C | C | C |  |
|  | *aph*6-Id |  | C | C |  | P^1^ | C | C | C |  |
| Beta-lactam | *bla*CMY-42 |  |  |  |  |  | P^4^ |  |  |  |
|  | *bla*CTX-M-15 |  |  |  |  |  |  |  |  | C |
|  | *bla*CTX-M-55 | C | P^5^ | C |  |  |  | P^2^ | P^3^ |  |
|  | *bla*SHV-2A |  |  |  |  | P^1^ |  |  |  |  |
|  | *bla*TEM-1 | P^6^ |  |  |  |  | C, P^4^ | C | C |  |
| Lincosamide | *inu*F |  |  |  |  |  |  |  | P |  |
| Macrolide | *mph*A | C |  |  |  |  | C | C | C |  |
| Phenicol | *flo*R |  |  | C |  |  |  |  | P^3^ |  |
| Quinolone | *qnr*S1 |  |  |  |  |  | P^4^ |  |  | C |
| Sulfonamide | *sul*1 | C | C |  |  | P^1^ | C | C | C |  |
|  | *sul*2 |  | C | C |  |  | C | C | C |  |
|  | *sul*3 |  |  |  |  |  |  |  | P^3^ |  |
| Tetracycline | *tet*A | C | P^5^ | C |  | P^1^ | C | P^2^ | P^3^ |  |
|  | *tet*B |  | C |  |  |  |  |  | C |  |
| Trimethoprim | *dfr*A17 | C | C |  |  |  | C | C | C |  |

^1^ pSHV2A plasmid in Fig. 3; ^2^ pCTXM55 plasmid in Fig. S4; ^3^ pCTXM55 plasmid in Fig. S5; ^4^ pTEM1 plasmid in Fig. S3; ^5^ pCTXM55 plasmid in Fig. S2; ^6^ pTEM1 plasmid in Fig. S1;

**Table S4.** Incompatibility groups of the plasmids within the nine resolved ESBL *E. coli* genomes.

| Isolate | Plasmid Name | Putative Incompatibility Group |
| --- | --- | --- |
| ESBL-B | pTEM1 (Fig. S1) | IncN |
| ESBL-C | pCTXM55 (Fig. S2) | IncF |
| ESBL-D | p1 | IncI |
|  | p2 | IncHI1 |
| ESBL-E | p1 | IncA/C |
|  | p2 | IncI |
|  | p3 | ND |
| ESBL-F | pSHV2A (Fig. 3) | IncF |
|  | p2 | IncHI1 |
| ESBL-G | p1 | IncF |
|  | pTEM1 (Fig. S3) | IncI |
| ESBL-I | p1 | IncF |
|  | pCTXM55 (Fig. S4) | IncI |
| ESBL-J | pCTXM55 (Fig. S5) | IncF |
| ESBL-K | p1 | IncI |

**Table S5.** Genotypes of transconjugant ESBL E. coli isolates. Plus sign (+) indicates the target antibiotic resistance genes was detected in all the six randomly selected colonies from a transconjugant selective plate. Negative sign (-) indicates the target antibiotic resistance genes were not detected in any of the six randomly selected colonies from a transconjugant selective plate.

| Donor isolate ID | Antibiotic resistance genes | | | | | | | | | |
| --- | --- | --- | --- | --- | --- | --- | --- | --- | --- | --- |
|  | *bla*CTX-M | *bla*TEM | *bla*SHV | *bla*CMY | *aad* | *aph* | *mph* | *sul*1 | *tet*A | *dfr*A |
| B | - | + | - | - | - | - | - | - | - | - |
| C | + | - | - | - | + | + | - | + | + | - |
| F | - | - | + | - | + | + | - | + | + | - |
| G | - | + | - | + | - | - | - | - | - | - |
| J | + | - | - | - | + | - | - | - | + | - |

**Table S6**. Alpha diversity indices of microbial genera and antibiotic resistance genes for the eleven wastewater samples

| Wastewater sample | |  | Microbial genera | | |  | Antibiotic resistance genes | | |
| --- | --- | --- | --- | --- | --- | --- | --- | --- | --- |
| ID | Type |  | Shannon diversity | Richness | Evenness |  | Shannon diversity | Richness | Evenness |
| A | Influent |  | 4.91 | 5,353 | 0.57 |  | 4.41 | 378 | 0.74 |
| B | Influent |  | 4.77 | 5,362 | 0.56 |  | 4.48 | 388 | 0.75 |
| C | Biosolids |  | 6.28 | 5,429 | 0.73 |  | 3.27 | 144 | 0.66 |
| D | Biosolids |  | 6.25 | 5,418 | 0.73 |  | 3.72 | 172 | 0.72 |
| E | Secondary |  | 6.20 | 5,478 | 0.72 |  | 3.89 | 219 | 0.72 |
| F | Effluent |  | 6.05 | 5,505 | 0.70 |  | 4.38 | 277 | 0.78 |
| G | Secondary |  | 6.16 | 5,524 | 0.72 |  | 4.16 | 250 | 0.75 |
| H | Biosolids |  | 6.32 | 5,521 | 0.73 |  | 3.88 | 194 | 0.74 |
| I | Effluent |  | 4.96 | 3,220 | 0.61 |  | 3.09 | 144 | 0.62 |
| J | Influent |  | 4.93 | 5,415 | 0.57 |  | 4.35 | 355 | 0.74 |
| K | Effluent |  | 5.65 | 5,088 | 0.66 |  | 3.20 | 215 | 0.60 |

**Table S7.** Abundance of antibiotic resistance genes in ESBL-producing E. coli-originating wastewater samples. The genes were present in the ESBL-producing E. coli isolates (as illustrated in Fig. 1 and Table S3)). Values are in units of gene copies/16S rRNA × 10^3^. Conditionally formatted with green for higher abundance values.

| Antibiotic resistance genes | Wastewater ID | | | | | | | | |
| --- | --- | --- | --- | --- | --- | --- | --- | --- | --- |
|  | B | C | D | E | F | G | I | J | K |
| *aac(3)-II* | 0.000 | 0.136 | 0.102 | 0.000 | 0.000 | 0.052 | 0.835 | 0.000 | 0.000 |
| *aac(3)-VI* | 0.010 | 0.000 | 0.000 | 0.000 | 0.000 | 0.000 | 0.000 | 0.000 | 0.000 |
| *aadA* | 13.875 | 2.916 | 4.254 | 7.578 | 17.501 | 10.646 | 1.702 | 18.626 | 6.196 |
| *aph(3')-I* | 0.264 | 0.000 | 0.000 | 0.000 | 0.524 | 0.000 | 0.039 | 0.642 | 0.000 |
| *bla*_CMY-42_ | 0.000 | 0.000 | 0.000 | 0.000 | 0.000 | 0.000 | 0.000 | 0.000 | 0.000 |
| *bla*_CTX-M-15_ | 0.000 | 0.000 | 0.000 | 0.000 | 0.000 | 0.000 | 0.000 | 0.000 | 0.000 |
| *bla*_CTX-M-55_ | 0.015 | 0.000 | 0.000 | 0.000 | 0.000 | 0.000 | 0.000 | 0.000 | 0.000 |
| *bla*_SHV-2A_ | 0.000 | 0.000 | 0.000 | 0.000 | 0.000 | 0.000 | 0.000 | 0.000 | 0.000 |
| *bla*_TEM-1_ | 0.226 | 0.000 | 0.000 | 0.035 | 0.183 | 0.000 | 0.000 | 0.048 | 0.054 |
| *dfrA17* | 0.028 | 0.000 | 0.000 | 0.000 | 0.000 | 0.000 | 0.055 | 0.000 | 0.169 |
| *floR* | 0.501 | 0.193 | 0.200 | 0.228 | 0.311 | 0.138 | 2.137 | 0.369 | 0.455 |
| *mph(A)* | 0.607 | 0.100 | 0.000 | 0.499 | 1.462 | 0.172 | 0.000 | 2.245 | 0.257 |
| *qacE∆1* | 7.905 | 1.132 | 2.328 | 7.529 | 12.303 | 12.186 | 4.313 | 19.658 | 9.559 |
| *qnrS* | 2.123 | 0.061 | 0.000 | 0.522 | 2.191 | 0.242 | 0.000 | 2.112 | 0.468 |
| *sul1* | 13.376 | 5.082 | 3.686 | 8.948 | 17.500 | 23.148 | 7.928 | 17.661 | 13.709 |
| *sul2* | 1.997 | 2.426 | 3.680 | 7.168 | 4.007 | 3.688 | 0.135 | 4.245 | 0.932 |
| *sul3* | 0.078 | 0.988 | 0.426 | 0.000 | 0.000 | 0.000 | 0.011 | 0.000 | 0.000 |
| *tet(A)* | 5.334 | 0.398 | 0.349 | 1.738 | 3.484 | 1.504 | 0.528 | 4.792 | 1.407 |
| *tet(B)* | 0.000 | 0.000 | 0.000 | 0.000 | 0.000 | 0.010 | 0.000 | 0.017 | 0.000 |

**Table S8.** ESBL-associated genes above 0.001 gene copies/16S rRNA abundance in wastewater samples.

| ESBL gene | Wastewater ID | | | | | | | | |
| --- | --- | --- | --- | --- | --- | --- | --- | --- | --- |
|  | B | C | D | E | F | G | I | J | K |
| *class A beta-lactamase* | 0.016 | 0.001 | 0.008 | 0.034 | 0.011 | 0.014 | 0.000 | 0.011 | 0.061 |
| *AER-1* | 0.002 | 0.000 | 0.000 | 0.013 | 0.001 | 0.002 | 0.000 | 0.006 | 0.001 |
| *CfxA2* | 0.011 | 0.000 | 0.000 | 0.005 | 0.006 | 0.010 | 0.000 | 0.009 | 0.003 |
| *OXA-2* | 0.007 | 0.000 | 0.002 | 0.003 | 0.006 | 0.004 | 0.000 | 0.009 | 0.004 |
| *OXA-10* | 0.007 | 0.000 | 0.000 | 0.002 | 0.004 | 0.002 | 0.000 | 0.005 | 0.001 |
| *class C beta-lactamase* | 0.003 | 0.000 | 0.000 | 0.000 | 0.003 | 0.000 | 0.000 | 0.001 | 0.000 |
| *OXA-333* | 0.002 | 0.000 | 0.000 | 0.000 | 0.001 | 0.000 | 0.000 | 0.000 | 0.000 |
| *CfxA3* | 0.002 | 0.000 | 0.000 | 0.001 | 0.001 | 0.002 | 0.000 | 0.001 | 0.001 |
| *OXA-119* | 0.000 | 0.000 | 0.001 | 0.002 | 0.002 | 0.001 | 0.000 | 0.001 | 0.000 |
| *penA* | 0.002 | 0.000 | 0.000 | 0.000 | 0.001 | 0.000 | 0.000 | 0.000 | 0.000 |
| *OXA-9* | 0.000 | 0.000 | 0.000 | 0.001 | 0.001 | 0.002 | 0.000 | 0.000 | 0.000 |
| *OXA-211* | 0.002 | 0.000 | 0.000 | 0.000 | 0.001 | 0.000 | 0.000 | 0.000 | 0.000 |
| *OXA-212* | 0.002 | 0.000 | 0.000 | 0.000 | 0.000 | 0.000 | 0.000 | 0.000 | 0.000 |
| *OXA-12* | 0.002 | 0.000 | 0.000 | 0.000 | 0.001 | 0.000 | 0.000 | 0.001 | 0.000 |
| *metallo-beta-lactamase* | 0.001 | 0.000 | 0.000 | 0.000 | 0.001 | 0.001 | 0.000 | 0.001 | 0.001 |
| *OXA-34* | 0.001 | 0.000 | 0.000 | 0.000 | 0.001 | 0.001 | 0.000 | 0.001 | 0.001 |
| *OXA-129* | 0.000 | 0.000 | 0.000 | 0.001 | 0.001 | 0.001 | 0.000 | 0.001 | 0.000 |
| *OXA-226* | 0.001 | 0.000 | 0.000 | 0.001 | 0.001 | 0.001 | 0.000 | 0.001 | 0.001 |
| *OXA-118* | 0.000 | 0.000 | 0.000 | 0.001 | 0.001 | 0.001 | 0.000 | 0.000 | 0.000 |
| *OXA-142* | 0.001 | 0.000 | 0.000 | 0.000 | 0.001 | 0.000 | 0.000 | 0.000 | 0.000 |
| *CARB-6* | 0.000 | 0.000 | 0.000 | 0.000 | 0.000 | 0.000 | 0.001 | 0.000 | 0.000 |
| *OXA-309* | 0.001 | 0.000 | 0.000 | 0.000 | 0.000 | 0.000 | 0.000 | 0.000 | 0.000 |


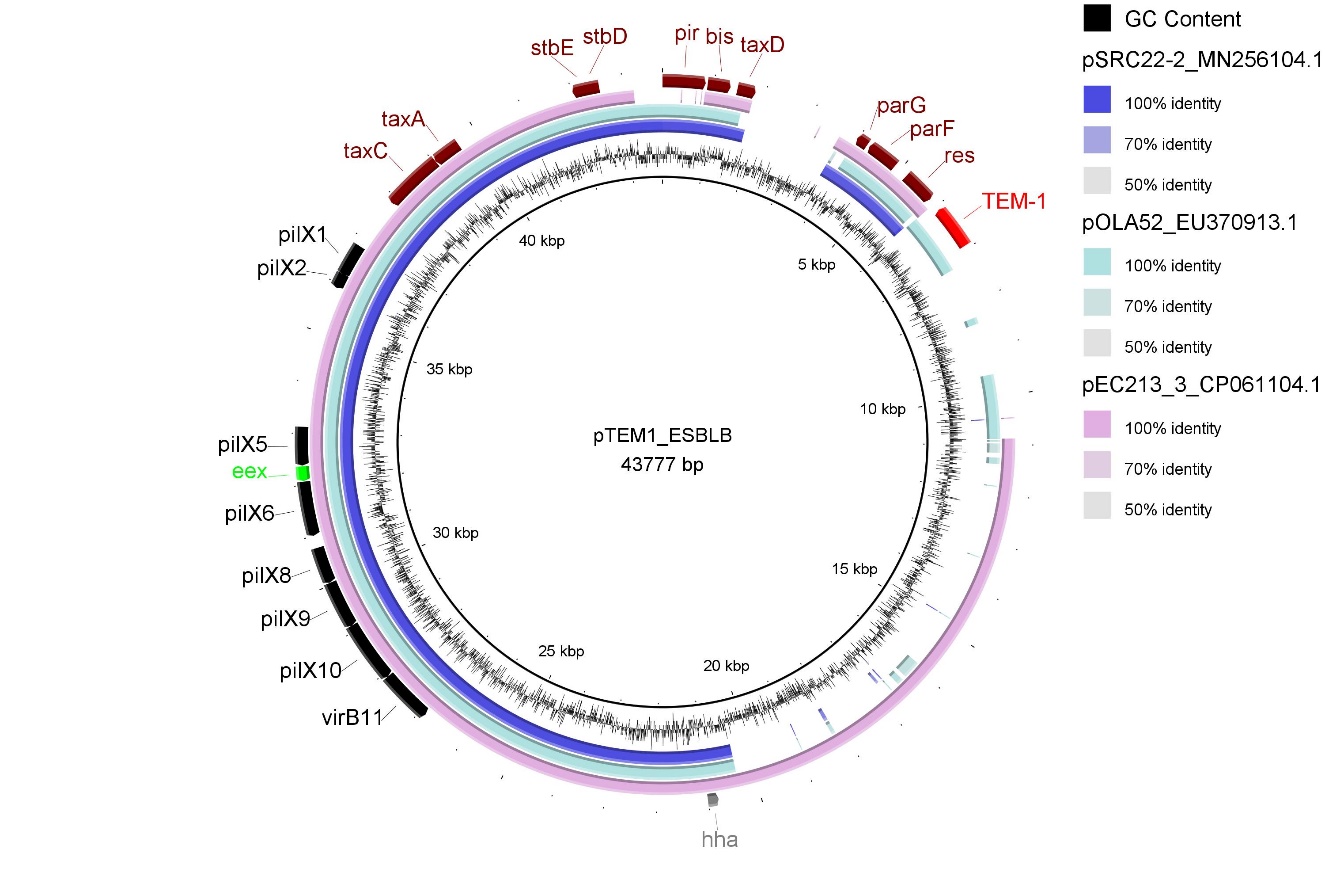


**FIG S1** Alignment of pTEM1 plasmid in ESBL-B E. coli isolate and closest BLAST hits (dark and light blue as well as pink rings) from the GenBank database. Gene labels are colored according to protein function: green – virulence factor, black – conjugation, maroon – plasmid replication/maintenance, red – antibiotic resistance gene, blue – mobile genetic element, and gold – mercury resistance.

.
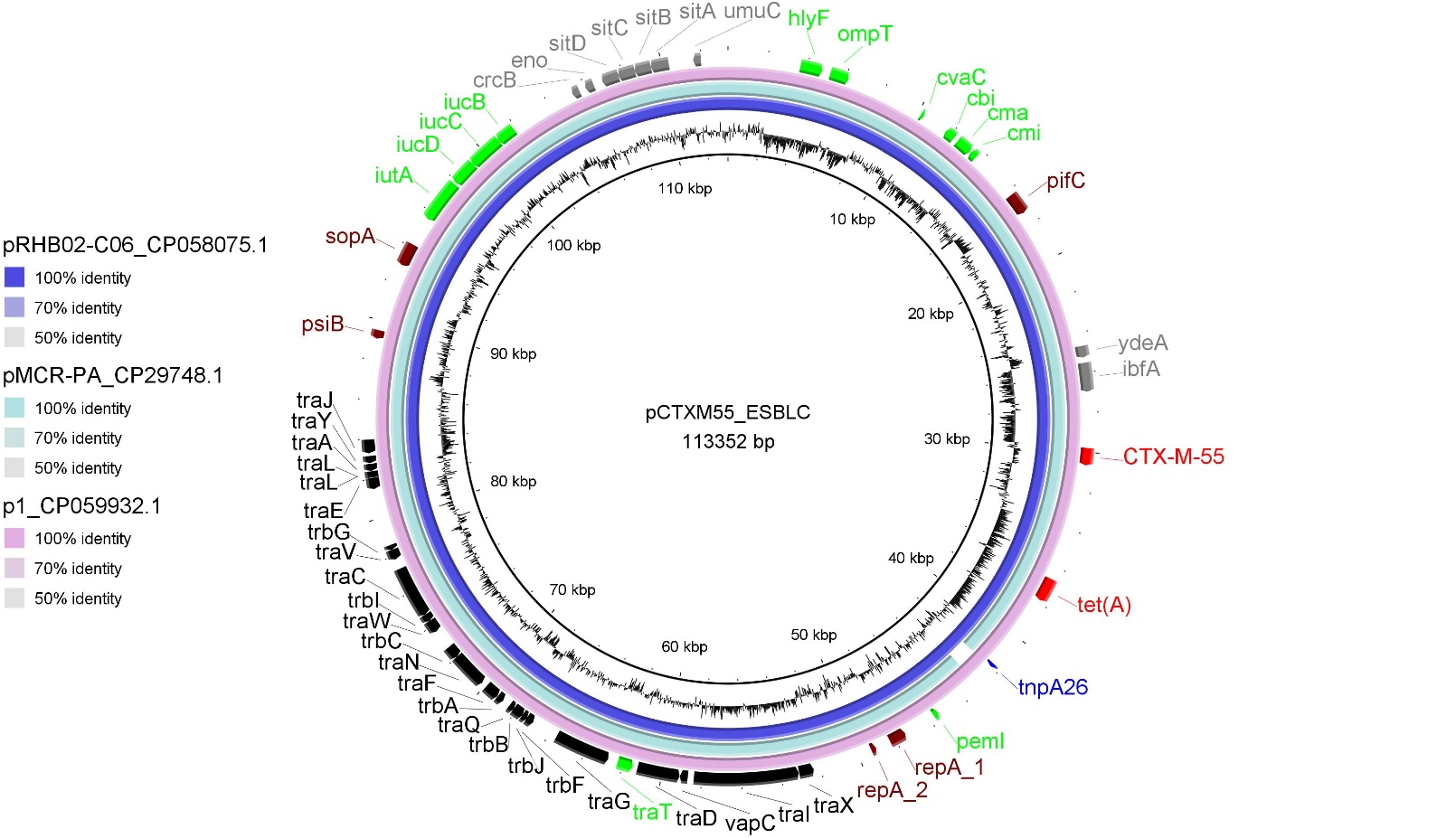


**FIG S2** Alignment of pCTXM55 plasmid in ESBL-C E. coli isolate and closest BLAST hits (dark and light blue as well as pink rings) from the GenBank database. Gene labels are colored according to protein function: green – virulence factor, black – conjugation, maroon – plasmid replication/maintenance, red – antibiotic resistance gene, blue – mobile genetic element, and gold – mercury resistance.


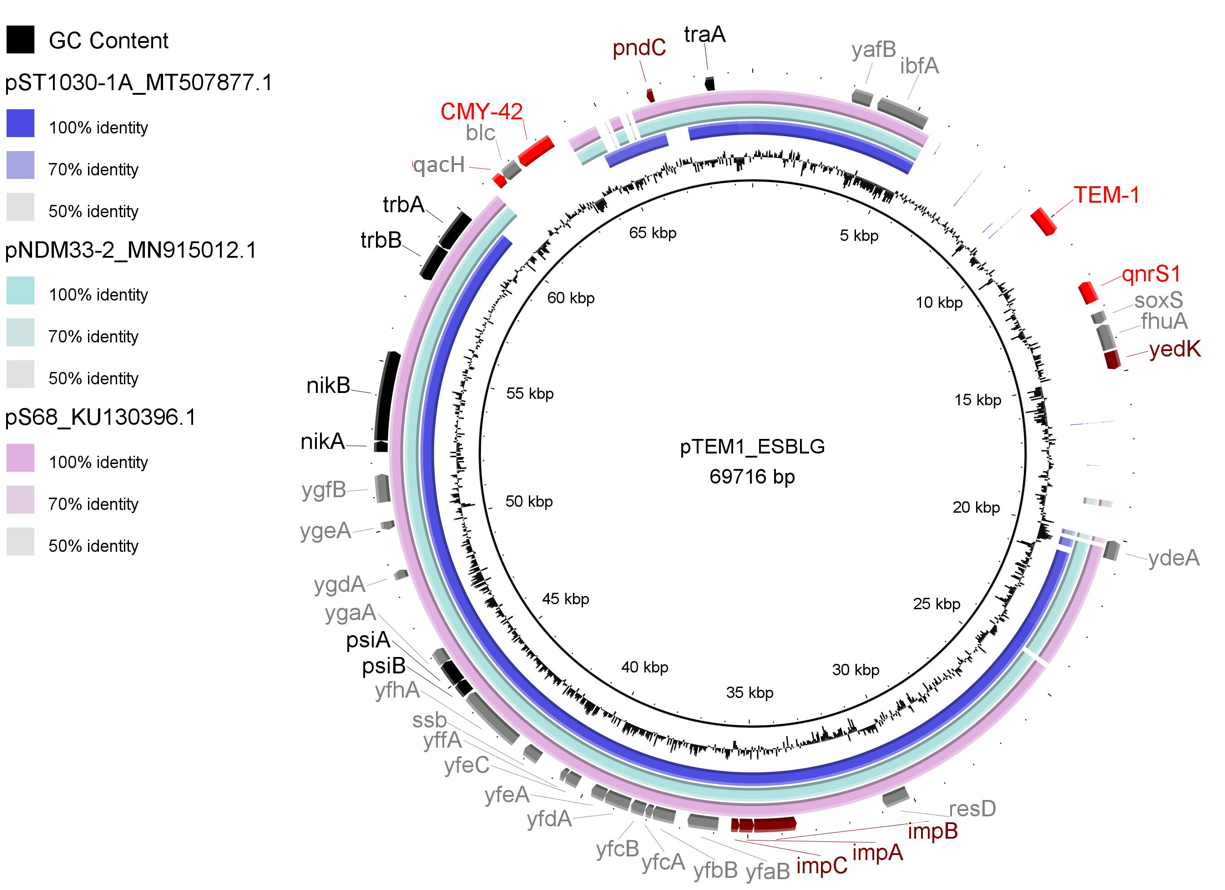


**FIG S3** Alignment of pTEM1 plasmid in ESBL-G E. coli isolate and closest BLAST hits (dark and light blue as well as pink rings) from the GenBank database. Gene labels are colored according to protein function: green – virulence factor, black – conjugation, maroon – plasmid replication/maintenance, red – antibiotic resistance gene, blue – mobile genetic element, and gold – mercury resistance.


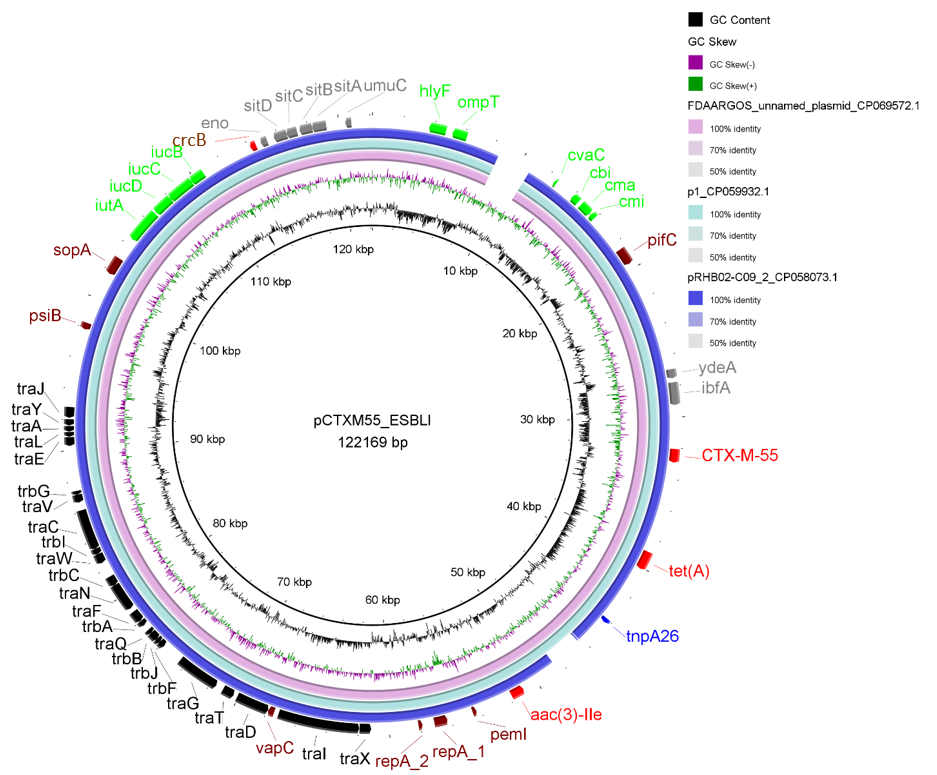


**FIG S4** Alignment of pCTXM55 plasmid in ESBL-I E. coli isolate and closest BLAST hits (dark and light blue as well as pink rings) from the GenBank database. Gene labels are colored according to protein function: green – virulence factor, black – conjugation, maroon – plasmid replication/maintenance, red – antibiotic resistance gene, blue – mobile genetic element, and gold – mercury resistance.


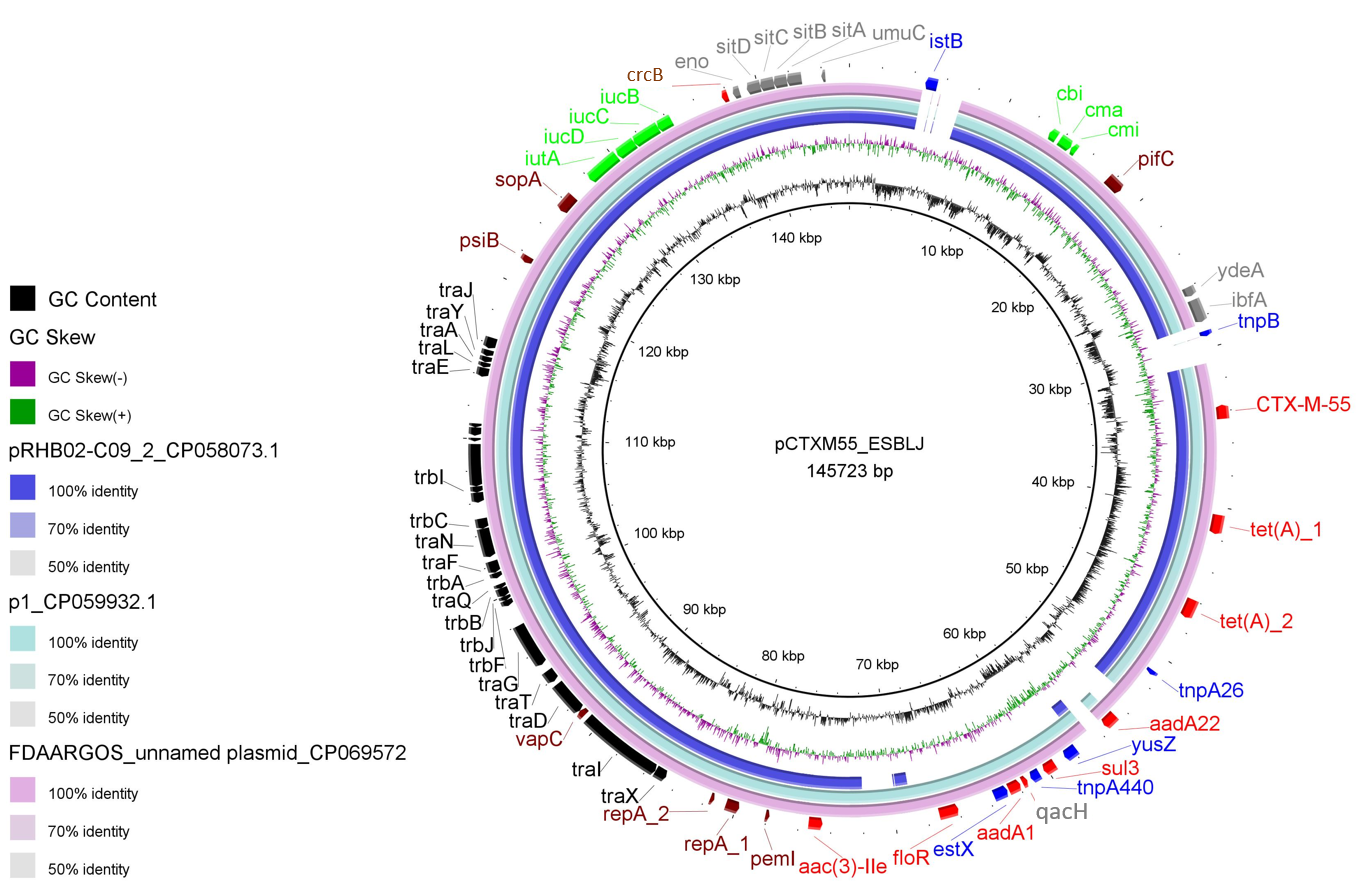


**FIG S5** Alignment of pCTXM55 plasmid in ESBL-J E. coli isolate and closest BLAST hits (dark and light blue as well as pink rings) from the GenBank database. Gene labels are colored according to protein function: green – virulence factor, black – conjugation, maroon – plasmid replication/maintenance, red – antibiotic resistance gene, blue – mobile genetic element, and gold – mercury resistance.

| a) Bray-Curtis dissimilarity scores for microbial genera | | | | | | |  |  |  |  |  |
| --- | --- | --- | --- | --- | --- | --- | --- | --- | --- | --- | --- |
| **B** | 0.22 |  |  |  |  |  |  |  |  | 0.10 | 0 |
| **C** | 0.62 | 0.63 |  |  |  |  |  |  |  |  | 0 |
| **D** | 0.60 | 0.61 | 0.24 |  |  |  |  |  |  | 0.50 | 1 |
| **E** | 0.56 | 0.59 | 0.41 | 0.40 |  |  |  |  |  |  | 1 |
| **F** | 0.48 | 0.50 | 0.44 | 0.44 | 0.25 |  |  |  |  | 0.90 | 1 |
| **G** | 0.44 | 0.47 | 0.44 | 0.42 | 0.29 | 0.24 |  |  |  |  |  |
| **H** | 0.61 | 0.61 | 0.15 | 0.17 | 0.40 | 0.42 | 0.42 |  |  |  |  |
| **I** | 0.68 | 0.73 | 0.67 | 0.67 | 0.62 | 0.64 | 0.63 | 0.66 |  |  |  |
| **J** | 0.33 | 0.30 | 0.60 | 0.57 | 0.57 | 0.49 | 0.44 | 0.58 | 0.72 |  |  |
| **K** | 0.53 | 0.59 | 0.51 | 0.48 | 0.41 | 0.42 | 0.42 | 0.49 | 0.65 | 0.55 |  |
|  | **A** | **B** | **C** | **D** | **E** | **F** | **G** | **H** | **I** | **J** |  |
|  |  |  |  |  |  |  |  |  |  |  |  |
| b) Bray-Curtis dissimilarity scores for antibiotic resistance genes | | | | | | | | |  |  |  |
| **B** | 0.12 |  |  |  |  |  |  |  |  |  |  |
| **C** | 0.79 | 0.79 |  |  |  |  |  |  |  |  |  |
| **D** | 0.64 | 0.65 | 0.32 |  |  |  |  |  |  |  |  |
| **E** | 0.49 | 0.51 | 0.69 | 0.55 |  |  |  |  |  |  |  |
| **F** | 0.26 | 0.29 | 0.76 | 0.60 | 0.36 |  |  |  |  |  |  |
| **G** | 0.39 | 0.42 | 0.70 | 0.53 | 0.31 | 0.26 |  |  |  |  |  |
| **H** | 0.55 | 0.56 | 0.41 | 0.21 | 0.51 | 0.49 | 0.46 |  |  |  |  |
| **I** | 0.74 | 0.74 | 0.71 | 0.66 | 0.65 | 0.71 | 0.70 | 0.64 |  |  |  |
| **J** | 0.32 | 0.35 | 0.76 | 0.60 | 0.43 | 0.27 | 0.34 | 0.51 | 0.71 |  |  |
| **K** | 0.52 | 0.54 | 0.75 | 0.65 | 0.39 | 0.49 | 0.47 | 0.59 | 0.64 | 0.45 |  |
|  | **A** | **B** | **C** | **D** | **E** | **F** | **G** | **H** | **I** | **J** |  |

**FIG S6** Bray-Curtis dissimilarity scores for (a) microbial genera and (b) antibiotic resistance genes in wastewater samples. Green indicates more similarity and red indicates more dissimilarity. Letters A-K indicate Sample IDs.


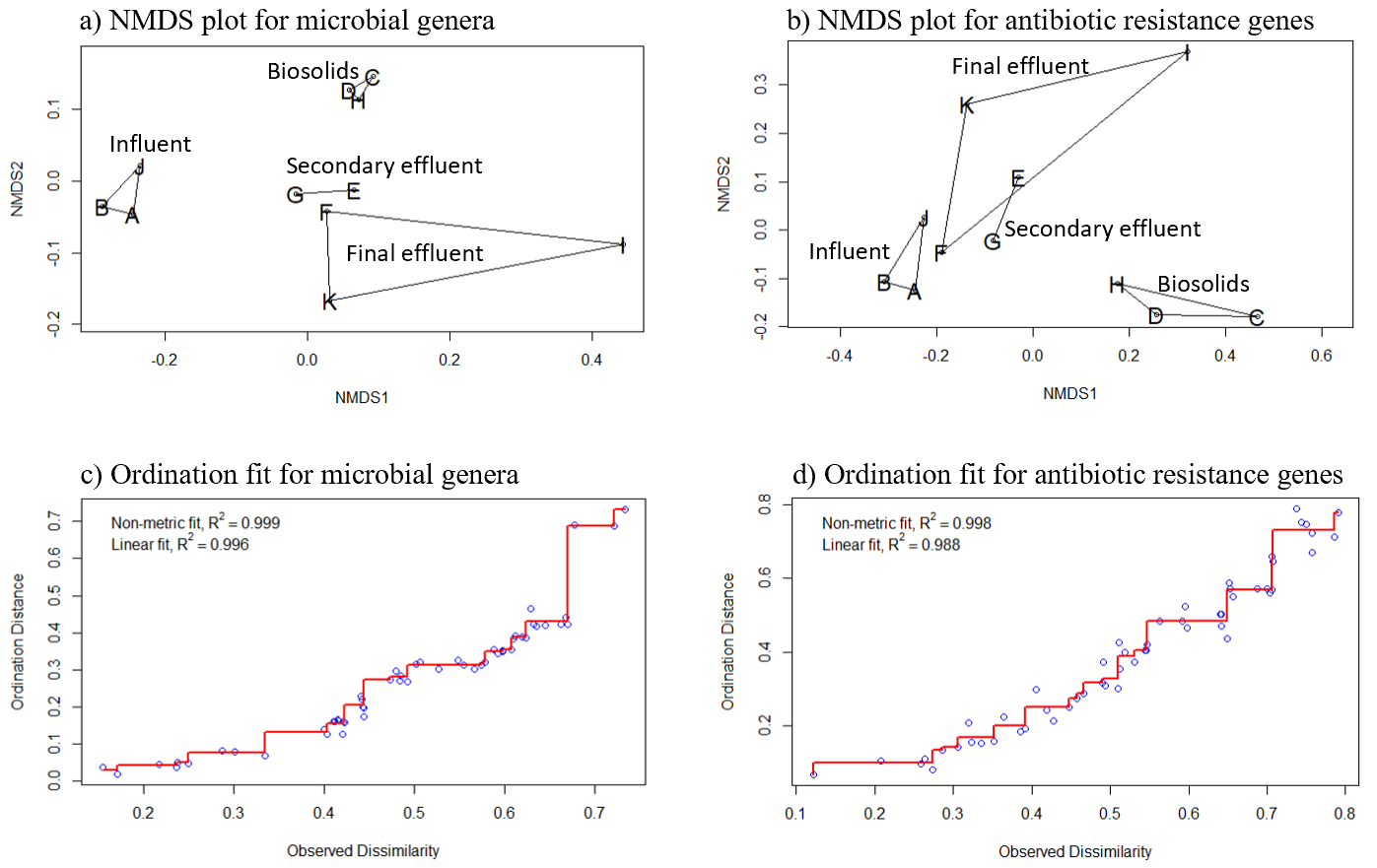


**FIG S7** NMDS plots (top) for (a) microbial genera and (b) antibiotic resistance genes, and ordination fits for (c) microbial general and (d) antibiotic resistance genes. In NMDS plots, letters A-K indicate Sample IDs and are connected by sample types with polygons.

**References**

1. Easler M, Cheney C, Johnson JD, Zadeh MK, Nguyen JN, Yiu SY, et al. Resistome characterization of extended-spectrum beta-lactamase (ESBL)-producing Escherichia coli isolated from wastewater treatment utilities in Oregon. Journal of Water and Health. 2022 Mar 21;20(4):670–9.

2. Momtaz H, Rahimi E, Moshkelani S. Molecular detection of antimicrobial resistance genes in E. coli isolated from slaughtered commercial chickens in Iran. Veterinarni Medicina. 2012 May 18;57(No. 4):193–7.

3. Dolejská M, Bierošová B, Kohoutová L, Literák I, Čížek A. Antibiotic-resistant Salmonella and Escherichia coli isolates with integrons and extended-spectrum beta-lactamases in surface water and sympatric black-headed gulls. Journal of Applied Microbiology. 2009;106(6):1941–50.

4. Guillaume G, Verbrugge D, Chasseur-Libotte ML, Moens W, Collard JM. PCR typing of tetracycline resistance determinants (Tet A–E) in Salmonella enterica serotype Hadar and in the microbial community of activated sludges from hospital and urban wastewater treatment facilities in Belgium. FEMS Microbiology Ecology. 2000 Apr 1;32(1):77–85.
